# A severe neurodevelopmental syndrome linked to a South Asian founder variant in the UFMylation adaptor *CDK5RAP3*

**DOI:** 10.1007/s00401-026-03017-2

**Published:** 2026-04-27

**Authors:** Michaela Yuen, Katharine Zhang, Rhett G. Marchant, Ryosuke Ishimura, Mark Graham, May Aung-Htut, Samantha Bryen, Rocio Rius, Lee Marshall, Nader Aryamanesh, Gregory Dziaduch, Himanshu Joshi, Ben Weisburd, Steve D. Wilton, Meredith Wilson, Russell Gear, Lucy Hennington, Stephanie Lau, Helen Doyle, Michael Krivanek, Richard J. Leventer, Susan M. White, Sarah A. Sandaradura, Masaaki Komatsu, Frances J. Evesson, Sandra T. Cooper

**Affiliations:** 1 Kids Neuroscience Centre, Kids Research, The Children’s Hospital at Westmead, Westmead, NSW 2145 Australia; 2 Functional Neuromics, Children’s Medical Research Institute, Westmead, NSW 2145 Australia; 3 Neuroscience, School of Medical Sciences, Faculty of Medicine and Health, The University of Sydney, Camperdown, NSW 2006 Australia; 4 Department of Physiology, Juntendo University Graduate School of Medicine, Tokyo, 113-8421 Japan; 5 Children’s Medical Research Institute, Faculty of Medicine and Health, The University of Sydney, Camperdown, NSW 2006 Australia; 6 Biomedical Proteomics, Children’s Medical Research Institute, Westmead, NSW 2145 Australia; 7 Personalised Medicine Centre, Health Futures Institute, Murdoch University, Murdoch, WA 6150 Australia; 8 Perron Institute for Neurological and Translational Science, Perth, WA 6009 Australia; 9 Centre for Population Genomics, Garvan Institute of Medical Research, UNSW Sydney, Darlinghurst, NSW 2010 Australia; 10 Centre for Population Genomics, Murdoch Children’s Research Institute, Parkville, VIC 3052 Australia; 11 Department of Paediatrics, University of Melbourne, Parkville, VIC 3010 Australia; 12 Bioinformatics Group, Children’s Medical Research Institute, Westmead, NSW 2145 Australia; 13 Broad Institute at MIT and Harvard, Boston, MA 02142 USA; 14 Department of Clinical Genetics, The Children’s Hospital at Westmead, Westmead, NSW 2145 Australia; 15 Murdoch Children’s Research Institute, Parkville, VIC 3052 Australia; 16 Clinical Genetics Department, The Mercy Hospital for Women, Heidelberg, VIC 3084 Australia; 17 Mercy Perinatal, Mercy Hospital for Women & Austin Health, Heidelberg, VIC 3084 Australia; 18 Alfred Health, Melbourne, VIC 3004 Australia; 19 Anatomical Pathology Department, Austin Health, Melbourne, Heidelberg, VIC 3084 Australia; 20 Department of Histopathology, The Children’s Hospital at Westmead, Westmead, NSW 2145 Australia; 21 Department of Neurology, The Royal Children’s Hospital Melbourne, Parkville, VIC 3052 Australia; 22 Department of Paediatrics, University of Melbourne, Melbourne, VIC 3010 Australia; 23 Victorian Clinical Genetics Services, Murdoch Children’s Research Institute, Parkville, VIC 3052 Australia

**Keywords:** Pontocerebellar hypoplasia (PCH), UFMylation, Splicing variant, Splice-switching antisense oligonucleotide, Neurodevelopmental disorder, Liver fibrosis, CDK5RAP3

## Abstract

We investigated the pathogenicity of a homozygous intronic variant in *CDK5RAP3*, a key UFMylation adapter, in three individuals from two unrelated families with a lethal neurodevelopmental disorder. *CDK5RAP3* variants have not been linked to human disorders to date; however, murine *Cdk5rap3* knockout is embryonic lethal and variants in five other UFMylation components cause severe neurodevelopmental conditions. A segregating homozygous variant, chr17(GRCh38):g.47974691G > A, *CDK5RAP3* NM_176096.3:c.334 + 243G > A, was identified by trio whole-genome and proband RNA sequencing in Family A and by trio whole-exome sequencing data reanalysis in Family B. Variant pathogenicity investigations included RT-PCR, Western blot, co-immunoprecipitation and (phospho)proteomics to assess transcript, protein and UFMylation complex effects. Antisense oligonucleotide-mediated rescue of *CDK5RAP3* expression combined with proteomics and phosphoproteomics defined the mechanistic impact of CDK5RAP3 deficiency and rescue in amniocytes from an affected individual. All three affected individuals showed foetal growth restriction, foetal akinesia, pontocerebellar hypoplasia, arthrogryposis and hepatic pathology. *CDK5RAP3* c.334 + 243G > A activates a cryptic donor splice-site causing pseudoexon/intron inclusion triggering nonsense-mediated decay and deficiency of full-length CDK5RAP3 (NP_788276.1), while potentially allowing retained expression of C-terminal alternative isoforms. Co-immunoprecipitation revealed only full-length CDK5RAP3 binds UFL1, whereas C-terminal isoforms cannot. Primary amniocytes showed CDK5RAP3 deficiency was associated with impaired UFMylation of known substrates, RPL26 and UFBP1. Proteomic and phosphoproteomic analyses revealed dysregulation of extracellular matrix organisation, cell adhesion, mitotic/genome stability pathways, cytoskeletal networks and neuronal guidance, which were reversed by restoration of canonical *CDK5RAP3* expression via splice-correcting antisense oligonucleotides. Phosphoproteomic data implicate CDK5RAP3 as an upstream regulator of UFL1 S462 phosphorylation, known to be regulated by Ataxia-telangiectasia mutated (ATM) signalling. Our findings provide strong evidence linking deficiency of full-length CDK5RAP3 to severe neurodevelopmental, liver and muscle dysfunction. This study further highlights the therapeutic potential of ASO-based deep-intronic splicing defect correction.

**Supplementary Information:**

The online version contains supplementary material available at 10.1007/s00401-026-03017-2.

## Introduction

Protein UFMylation is a reversible post-translational modification analogous to ubiquitination, involving covalent attachment of UFM1 (ubiquitin-fold modifier 1) to substrate proteins [36]. UFMylation regulates key cellular functions, including endoplasmic reticulum (ER) stress response, autophagy, cell cycle and protein quality control [reviewed in 37, 38]. Protein UFMylation is achieved through an evolutionarily conserved 3-step enzymatic cascade beginning with UFM1 activation through C-terminal cleavage by UFM1-specific proteases (UfSPs) exposing a C-terminal glycine [32, 48]. Activated UFM1 forms a thioester bond with the E1-like enzyme UBA5 (Cys250), is transferred to the E2-like enzyme UFC1 (Cys116) and is finally covalently conjugated to substrate lysines by the UFM1 E3-like ligase complex comprising UFL1-UFBP1-CDK5RAP3 [56].

Recently, pathogenic variants in several genes encoding UFMylation pathway proteins have been linked to recessive neurodegenerative and neurodevelopmental disorders in humans, including *UFM1* [27], *UFSP2* [66], *UBA5* [51], *UFC1* [3] and *DDRGK1* (aka *UFBP1*[20]). Disease onset and severity vary between individuals and genes. Common clinical features include global developmental delay, epileptic encephalopathy, ataxia, microcephaly and brain pathology such as white matter changes, thin corpus callosum and cerebellar atrophy (Table S1). *UFSP2* and *DDRGK1* variants can also lead to dominant or recessive forms of skeletal dysplasia, respectively [18, 20, 24, 46, 66, 74]. Disorders associated with *UBA5* [4, 13, 51] and *UFM1* [27, 52] can be severe, presenting with fatal infantile epileptic encephalopathy or congenital neuropathy [8].

CDK5 regulatory subunit-associated protein 3 (CDK5RAP3, MIM#: 608202), a key UFMylation substrate adaptor [69], has no previous links to human disease. Here, we present compelling evidence connecting biallelic *CDK5RAP3* variants with a lethal neurodevelopmental disease in three individuals from two unrelated families—further highlighting a vital role of CDK5RAP3 in human neurodevelopment with suggested functions in regulation of UFL1 phosphorylation, extracellular matrix (ECM), cell adhesion and cell cycle regulation.

## Methods

### Ethics and patient consent

Informed, written consent was obtained from all study participants or their legal guardians. Family A was recruited through Kid’s Neurobank (later transitioned to the “Answers for Rare Disease” study) approved by the Human Research Ethics Committee of the Children’s Hospital at Westmead, Australia (2019/ETH11736; 2023/ETH02465). Family B was enrolled in the Rare Diseases Now study approved by the Royal Children’s Hospital Human Research Ethics Committee (HREC/67401/RCHM-2020).

### Massively parallel sequencing, pathogenic genetic variant discovery and aberrant pre-mRNA splicing predictions

Family A: DNA from whole blood (AII-1, AI-1, AI-2), buccal swabs (healthy siblings AII-2, AII-4) and amniocytes (AII-3) was used. Trio exome and genome sequencing (AI-1, AI-2, AII-1), and singleton skeletal muscle RNA sequencing (RNA-seq; AII-1), were performed by the Broad Institute (MIT and Harvard University, USA) as described [14]. Family A trio exome/genome data were analysed in seqr [53] and using a custom splicing variant detection pipeline [45]. Population allele frequencies were sourced from gnomAD v2.1.1 [33] via BigQuery (Google, Mountain View, CA, USA). Filtering was performed in R (v4.0.1; The R Foundation, Vienna, Austria) within RStudio (v1.1.463; Boston, MA, USA), retaining segregating variants with minor allele frequency < 0.01, SpliceAI [30] delta-scores ≥ 0.10 (variants were annotated with precomputed SpliceAI scores using bcftools (v1.9)). Prioritised variants were manually assessed in RNA-seq data using the Integrative Genomics Viewer (IGV; v2.10; Broad Institute, Boston, MA, USA) and splice-outliers were detected using FRASER (v1.0.2) [47] relative to 50 control skeletal muscle RNA-seq datasets from GTEx [9]. FRASER splice-outliers located within 250-bp of curated variants were manually scrutinised for aberrant splicing using IGV. Gene prioritisation for analysis was (1) neuromuscular/neurological genes, (2) other clinically relevant Mendelian genes [15], (3) murine essential genes not yet linked to a human disorder [16], and (4) all remaining genes. Segregation of NM_176096.3:c.334 + 243G > A was confirmed by PCR amplification of a 297-bp region (all PCR primer and amplification details are in Table S2). PCR products were treated with Exonuclease I and Shrimp Alkaline Phosphatase (New England Biolabs), bi-directionally Sanger sequenced (Australian Genome Research Facility, Sydney), and analysed in Sequencher 4.8 (Gene Codes Corporation, Ann Arbor, MI, USA).

Family B: Exome sequencing data processing was performed by the Centre for Population Genomics (CPG) CaRDinal platform following the DRAGEN GATK best practices pipeline. Reads were aligned to the hg38 reference genome using Dragmap (v1.3.0). Cohort-wide joint calling of single nucleotide variants (SNVs) and small insertion/deletion (indel) variants was performed using GATK HaplotypeCaller (v4.2.6.1) with “--dragen-mode” enabled. Variants were annotated using VEP 110, and analysed in CPG’s CaRDinal deployment of seqr. Sample gender and relatedness quality checks were performed using Somalier (v0.2.15) [55].

### Reverse transcription (RT) and quantitative (q) PCR

For RT-PCR, cDNA synthesis and PCR were performed as described [72]. For qPCR, cDNA was synthesised using the qScript Ultra Flex kit (Quantabio, Beverly, MA, USA) and Oligo(dT) primers. qPCR was performed using PowerUp SYBR Green master mix on a QuantStudio™ 6 Pro Real-Time PCR system (Thermo Fisher Scientific, Waltham, MA, USA). *CDK5RAP3* transcript expression levels were normalised to the geometric mean of three reference genes (*GAPDH*, *HPRT1*, and *RPLP0*) and plotted as relative to control samples, as per [61]. *XBP1* splicing was assessed as described [22].

### Haplotype analysis

The size of the runs of homozygosity (ROH) containing the NM_176096.3:c.334 + 243G > A variant was compared across exome data from six individuals (AII-1, AI-1, AI-2, BII-1, BI-1, BI-2). Since AII-1 had the smaller ROH, variants called within this region in Family A exome data were filtered for in Family B exome data (GRCh38 chr17:45,983,659–49,424,990). Only variants called in all six individuals with genotype quality > 35 were retained. Genome data from AII-1, AI-1, and AI-2 were later analysed to refine the ROH boundaries.

### Western blot

Lysates were prepared as described [7]. 5 μg (tissues) or 15 μg (cells) of protein was electrophoresed on 1 mm 4–12% Bolt™ Bis–Tris Mini Protein Gels (Invitrogen, Thermo Fisher Scientific, Waltham, MA, USA) followed by transfer and antibody probing as described [7]. Primary antibodies were rabbit anti-CDK5RAP3 (PA5-89007, 1:2000, Thermo Fisher Scientific, Waltham, MA, USA; HPA022141, 1:2500, Sigma-Aldrich, St. Louis, MO, USA), rabbit anti-α-actinin (4B2; gift from A. Beggs; Boston Children’s Hospital, Boston, MA, USA), mouse anti-GAPDH (MAB374, 1:10,000, Merck Millipore, Burlington, MA, USA). Fluorescent secondary antibodies were anti-mouse IgG IRDye 800CW and anti-rabbit IgG IRDye 680CW (1:15,000), and fluorescence imaging was performed on the Odyssey XFC (all from LI-COR Biosciences, Lincoln, NE, USA).

### Co-immunoprecipitation

FLAG-tagged CDK5RAP3 wild-type (wt), ORF3, or ORF1 were expressed in *CDK5RAP3*-knockout HEK293T cells. Twenty-four hours post-transfection, cells were lysed as described [28] and 200 μl of lysate was incubated with 200 μl IP buffer plus 10 μl anti-FLAG M2 Affinity Agarose Gel (A2220, Merck Millipore, Burlington, MA, USA) with rotation overnight at 4 °C. Immunoprecipitates were washed five times with ice-cold IP buffer and eluted by boiling for 5 min in SDS sample buffer containing 1 M DTT. Proteins were resolved by SDS–polyacrylamide gel electrophoresis (SDS-PAGE), transferred to a polyvinylidene difluoride (PVDF) membrane (IPVH00010, Merck Millipore, Burlington, MA, USA) and probed with anti-UFL1 (A303-456A; Bethyl Laboratories, Montgomery, TX, USA; 1:1000), anti-CDK5RAP3 (H00080279-M01; Novus Biologicals, Englewood, CO, USA; 1:500), and anti-FLAG (M185-3 L, Medical & Biological Laboratories, Tokyo, Japan; 1:1000) antibodies. Horseradish peroxidase-conjugated goat anti-mouse or goat anti-rabbit immunoglobulin G (IgG, H + L, 1:10,000; 115-035-166 and 111-035-144, Jackson ImmunoResearch Laboratories Inc., West Grove, PA, USA) was used for detection by chemiluminescence.

### Primary amniocyte culture and antisense oligonucleotide (ASO) transfection

Primary amniocytes derived from AII-3 and healthy control 4 (C4) were cultured in AmnioMAX-II Complete Medium (Thermo Fisher Scientific, Waltham, MA, USA). Cells were treated with 100 μg/ml cycloheximide (CHX) 5 h before harvesting for RNA extraction to inhibit nonsense-mediated decay (NMD).

Cryptic pseudoexon (PE) donor splice-site targeting (ASO-T) and scrambled control (ASO-S) ASOs were synthesised as phosphorodiamidate morpholino oligonucleotides (PMO, Gene-Tools, LLC, Philomath, OR, USA, Table S2). 1 × 10^6^ amniocytes were electroporated with 20 µM ASOs in triplicate using the Neon NXT Electroporation System (2 × 20 ms 1300 V pulses; Thermo Fisher Scientific, Waltham, MA, USA), seeded into 10 cm dishes and harvested 72 h after electroporation.

### Liquid chromatography tandem mass tagging spectrometry (TMT LC–MS/MS)

Lysates were prepared as in Ref. [21], then reduced, alkylated, precipitated and digested with a LysC-trypsin mixture as described [62]. Samples were labelled with TMTpro reagent (~ 100 μg each, Lot XA340093, Thermo Fisher Scientific, Waltham, MA, USA) according to manufacturer’s instructions. Samples were combined, desalted using a solid-phase extraction cartridge (Sep-Pak C19 200 mg; Waters Corporation, Milford, MA, USA) and enrichment of phosphopeptides was performed as described [21]. Phosphopeptide-enriched and de-enriched samples were separately applied to hydrophobic liquid ion chromatography (HILIC) using a 5 μm TSKGel Amide 80 column (Tosoh Bioscience, Tokyo, Japan) essentially as described [21], except that fractions were collected every 60 s. LC–MS/MS was performed as in Ref. [62]; except for phosphopeptides, the gradient was from 5% buffer B to 25% buffer B in 74 min, the MS/MS ion time was increased to 115 ms, and the collision energy was 34.

### Proteome and phosphoproteome data analysis

Comprehensive proteomic and phosphoproteomic data-processing workflows are described in full in the Supplementary Methods.

### Proteomics and phosphoproteomics data filtering to obtain high-confidence CDK5RAP3-responsive proteins and pathways

(Phospho)proteomics datasets were filtered to identify a list of proteins/phosphosites significantly altered between primary amniocytes derived from AII-3 and control C4 (|log2FC|≥ 0.2*, and q-*value ≤ 0.05) in ‘Proteomics Dataset 1’ (proteomics data only), or the comparisons ASO-S-treated C4 vs ASO-S-treated AII-3 or ASO-T-treated C4 vs ASO-S-treated AII-3 in ‘Proteomics Dataset 2’ and ‘Phosphoproteomics Dataset’. From this list, we selected proteins restored by ASO-T, defined as (1) significantly altered between Proband_T_ and Proband_S_ samples (|log2FC|≥ 0.2, *q*-value ≤ 0.05), and (2) showing a *z*-score shift towards ASO-S or ASO-T-treated C4. A more permissive log2FC-threshold was used here to capture proteins only partially restored. Finally, proteins/phosphosites also altered between ASO-T and ASO-S-treated C4 were excluded (ASO-T off-target effects). Pathway analysis was performed on this ‘Rescue Set’ of restored, CDK5RAP3-responsive proteins as described in the Supplementary Methods.

## Results

We describe two families comprising three individuals affected with a severe neurodevelopmental disorder. Parents in both families are healthy and unrelated; the lack of relatedness was confirmed with Somalier [55] (relatedness score between parents of Family A: -0.014, and between parents of Family B: 0.007, cutoff < 0.05). Neither family reported a history of neuromuscular disorders, although the maternal grandmother of Family A had recurrent pregnancy loss of unknown aetiology. Both families are Nepalese: Family A is reportedly of South Asian/Indo-Aryan descent while Family B originates from Eastern Nepal with no known Indian heritage.

### Case report—family A

Proband AII-1 (male) was the first pregnancy for mother AI-2, complicated by insulin-treated gestational diabetes. A first trimester screen showed low aneuploidy risk and the morphology ultrasound was normal. A growth ultrasound at 31/40 weeks revealed foetal growth restriction (estimated foetal weight < 5th percentile) and small cerebellum (transcerebellar diameter < 5th percentile). A TORCH screen for antenatal infections and a foetal echocardiogram were normal. Reduced foetal movements were noted for several weeks prior to delivery.

Following spontaneous labour onset, AII-1 was delivered via caesarean section at 33 + 2/40 weeks due to foetal distress and breech presentation. Birth weight, head circumference and length were 1238 g (< 3rd percentile), 28.2 cm (7th percentile), and 41.5 cm (25th percentile), respectively. Abnormal hip and knee position was noted, with suspected congenital hip dislocation. APGAR scores were low (3, 5 and 6 at 1, 5 and 10 min, respectively), requiring ventilatory support and neonatal intensive care. Chest X-ray showed lung hypoplasia, and a single dose of surfactant and caffeine was administered.

Bedside cardiac ultrasound showed a structurally normal heart with normal outflow tracts and a bidirectional patent ductus arteriosus. Suspected late-onset sepsis (coagulase-negative Staphylococcus) was treated with antibiotics. AII-1 received phototherapy for physiological jaundice (bilirubin 168 μmol/L), albumin for hypoalbuminaemia (11 g/L) and a single packed red cell transfusion for anaemia with persistent borderline thrombocytopenia (platelet count 79–120 × 10^9^/L).

**Fig. 1:**
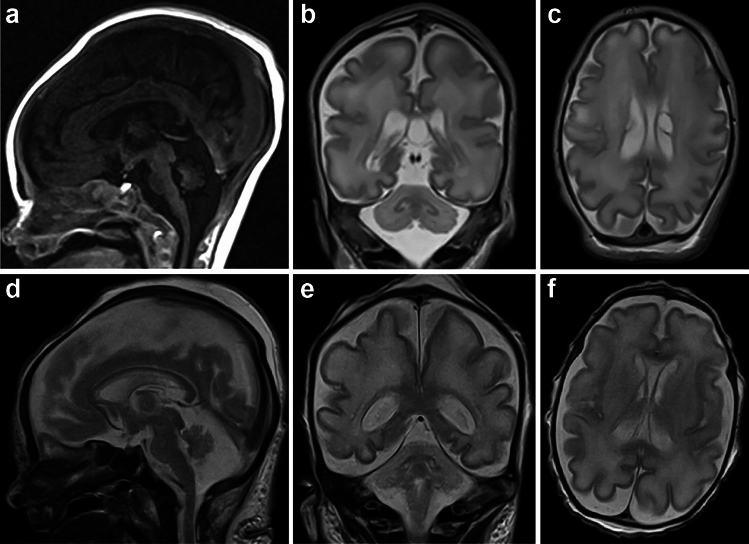
MRI of proband AII-1 and BII-1. **a** Midline sagittal FLAIR, **b** coronal T2 and **c** axial T2-weighted brain MRI of AII-1 at 7 days of age (corrected gestational age of 35/40 weeks), demonstrated a mildly small pons and pan-cerebellar hypoplasia. A cavum septum pellucidum and vergae, along with bilateral choroid plexus cysts, are visible in (**c**) (left: 9 × 6 mm; right: 6 × 3 mm; anteroposterior × transverse; mild gliotic lining of cysts observed during autopsy). **d** Sagittal, **e** coronal and **f** axial T2-weighted brain MRI of BII-1 at 12 days of age (corrected gestational age of 33 + 5/40 weeks) showing cerebellar vermian height, width and transcerebellar diameter are all more than 3 standard deviations below the mean for a 33-week-old corrected age neonate

Initial postnatal metabolic tests, including very long chain fatty acids, transferrin isoforms, lysosomal enzymes, glycine and ornithine, were normal, as were electromyography and ophthalmological assessment. Creatine kinase (CK) was elevated (798 U/L, single measurement, normal < 180 U/L). Skeletal survey showed gracile long bones. BRAINZ monitoring and electroencephalogram (EEG) at corrected age of 35/40 weeks’ gestation showed diffuse cerebral dysfunction and EEG seizures, which were treated with phenobarbitone. Repeat EEG 1 week later confirmed findings. Cranial ultrasound suggested lissencephaly. Brain magnetic resonance imaging (MRI) showed a hypoplastic cerebellum (transverse diameter < 2 SD below normal), cerebellar vermis, and pons; bilateral choroid plexus cysts were noted (Fig. 1a–c). Spine MRI found no cord abnormalities but suggested an epidural hematoma without spinal cord compression.

Neurologically, AII-1 had limited respiratory effort, minimal spontaneous antigravity movements, brisk reflexes with clonus, thin overlapping fingers, and arthrogryposis with finger, elbow, hip, knee and ankle contractures. Knees were fixed in hyperextension and ankles were in dorsiflexion. Given persistent ventilator dependence and poor prognosis, intensive care was redirected to palliation and AII-1 died at 33 days postnatal (corrected gestational age 37 + 5/40 weeks).

After the birth of a healthy male sibling (AII-2), the third pregnancy (AII-3, female) showed growth restriction and clenched hands at 20 + 5/40 weeks. Continuing growth restriction, cerebellar hypoplasia and multiple upper and lower joint contractures were confirmed at 25 + 4/40 weeks, and amniocentesis was performed. Given phenotypic resemblance to AII-1 and poor prognosis, the pregnancy was terminated at 26 + 6/40 weeks’ gestation.

Autopsy of AII-1 and AII-3 showed global, symmetric growth restriction. AII-1 weighed 1975 g at 37 + 5/40 weeks’ gestation, (expected for 33–34 weeks) and AII-3 weighed 602 g at 26 + 6/40 weeks’ gestation (expected 894 ± 135 g). Internal organs were small but normally positioned and formed (Table S3).

**Fig. 2:**
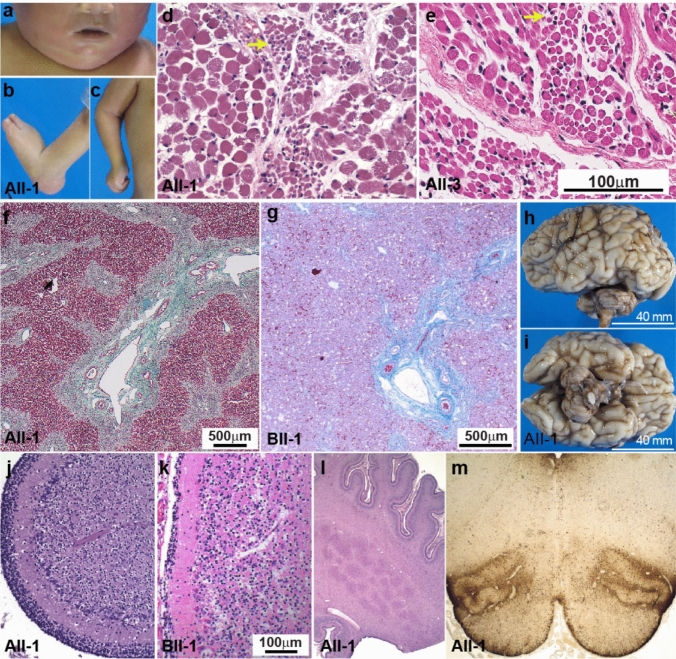
Pathology and histopathology of Family A and B. **a** Facial features of AII-1 at a corrected age 37 + 5/40 weeks’ gestation included micrognathia, a narrow mouth, and a high, narrow arched palate. **b**, **c** Fixed deformities of upper and lower limbs were evident; elbows mildly flexed, wrists hyperextended and fixed, fingers flexed mainly at the metacarpophalangeal and proximal interphalangeal joints, fisted hands with adducted thumbs, and boutonnière deformity of the 3rd and 4th fingers. Lower limbs show hypoflexed hips with congenital dislocation, fixed flexion deformities of the feet and ankles, and bilateral club and rocker-bottom feet. H&E-stained skeletal muscle of **d** AII-1 (psoas) and **e** AII-3 showed marked variability in myofibre diameter with both atrophic (yellow arrow) and hypertrophic fibres [2.5–22.0 μm in AII-1; 7.5–30.9 μm in AII-3; normal ~ 7 μm at 20/40 weeks’ gestation; [1], and internalised nuclei (AII-1: not quantified; AII-3: 10% of fibres). Scale bar for E and F is in F. Liver Masson Trichrome staining of **f** AII-1 and **g** BII-1 (40 × magnification). Liver histology of AII-1 showed irregular portal fibrosis, mild intrahepatocytic and bile canalicular cholestasis. Liver histology of BII-1 showed acute canalicular cholestasis with bile duct plugging, mild portal inflammation with ductular reaction and portal/periportal fibrosis with focal portal-portal bridging. **h** Lateral and **i** inferior view of the brain of AII-1 showed symmetrical cerebral hemispheres with sulcal and gyral development appropriate to gestational age. Notably, there is reduced size of the brainstem (2.5 g; 1.3% of total brain weight, normal: 2.4%) and cerebellum (4 g; 2.1% of brain weight, normal: 4.1%). H&E of the cerebellar cortex of **j** AII-1 and **k** BII-1 (200 × magnification) showing preserved lamination with normal external granular and molecular layers but a reduced number of Purkinje cells. **l** The dentate nucleus of AII-1 had a simplified, nodular architecture, reduced neuronal staining and increased surrounding glial cells. **m** The inferior olives showed poor demarcation from adjacent tissue, reduced neuronal staining and somewhat increased glial cell number on GFAP stain

Both siblings had distinctive facial features including high-arched palates and retro/micrognathia (AII-1 is shown in Fig. 2a; AII-3 had a downturned mouth and protuberant globes, not shown). Arthrogryposis with fixed upper and lower limb joint deformities was present in both siblings (Fig. 2b, c showing AII-1). Congenital hip dislocation was seen in AII-1 and hip adduction in AII-3.

Muscle atrophy was noted, more prominently affecting distal limbs in AII-3. Most muscles appeared macroscopically normal, but in AII-1, the quadriceps was fatty and ill-defined. Histology showed areas of myofibre diameter variability and an increased number of internal nuclei in both siblings, and angulated fibres in AII-3 (Fig. 2d, e).

Liver pathology in both siblings included a macroscopically fibrous appearance with portal fibrosis, ductular reaction (liver-injury-induced reactive bile duct proliferation), extramedullary haematopoiesis, and a mixed chronic inflammatory infiltrate (Fig. 2f). AII-1 showed mild intrahepatocytic and bile canalicular cholestasis, with bile ducts occasionally plugged (Fig. 2f). Early cholestasis and bile plugs were also seen in AII-3.

Brain weights were reduced: 190 g in AII-1 and 91.9 g in AII-3, compared to expected weights of 343.6 ± 37.9 g and 124.9 ± 18.2 g, respectively. Although the brainstem and cerebellum were macroscopically relatively normal, they were reduced in size (Fig. 2h, i). The cerebellar cortex appeared normal, but Purkinje cells were reduced or lacking (Fig. 2j showing AII-1). The dentate nucleus and inferior olivary nucleus (olives) were abnormally simplified in both siblings (Fig. 2l, m**)**, with olives showing minimal convolution and a simplified “U” shape (Fig. 2m**)**. AII-3 showed multiple discontinuous peripheral nodules and AII-1 showed fewer neurons and an increased number of glial cells surrounding the dentate on GFAP staining (Fig. 2m**)**. Brain pathology raised the possibility of pontocerebellar hypoplasia (PCH), particularly olivopontocerebellar hypoplasia (OPCH).

### Case report—family B

The affected child (BII-1) was the first pregnancy for his mother (BI-2) from a naturally conceived dichorionic, diamniotic twin pregnancy, with foetal demise of the co-twin at ~ 10 weeks. A 22-week ultrasound was unremarkable; however, foetal growth restriction (< 5th percentile) was evident by 31/40 weeks. Ongoing severe growth restriction, abnormal Doppler ultrasound and reduced foetal movement prompted an emergency Caesarean section delivery at 32/40 weeks’ gestation. Birth weight was 1.2 kg (5th percentile), head circumference was at the 3rd percentile, and length < 5th percentile. BII-1 was born in poor condition with hypotonia. Minimal spontaneous movements and respiratory effort (APGAR scores: 1 and 2) necessitated intubation and ventilation.

BII-1 had posteriorly rotated ears, synophrys, a broad nasal bridge, a low anterior hairline, and bilateral single palmar creases. Hands were clenched with camptodactyly and overlapping fingers. He had fixed talipes with overlapping toes. Both hips were dislocated. A small phallus and small testes were noted. Renal ultrasound showed mild bilateral pelvicalyceal dilatation. Echocardiogram showed right heart dilatation, mild tricuspid regurgitation, pulmonary hypertension, and a patent ductus arteriosus. Cranial ultrasound revealed a markedly small/hypoplastic cerebellum (<< 5th percentile) and right-sided grade I intraventricular haemorrhage. Brain MRI, though limited by artefact, showed PCH (Fig. 1d–f).

Ventilation requirements remained high over 6 weeks, progressing from conventional ventilation to high-frequency ventilation: all attempts at weaning were unsuccessful. He had marked tracheobronchomalacia. Care was redirected to palliation and BII-1 died on day 45 (corrected age: 38/40 weeks’ gestation).

Autopsy of BII-1 confirmed growth restriction (weight: < 5th percentile, normal length) and a markedly small brain (220 g; normal range 368.91 ± 39 g). Organ weights (heart, liver, thymus, adrenal glands) were < 5th percentile. Abnormal ear morphology, fixed foot contractures with external rotation and rocker-bottom appearance, loosely clenched hands, micropenis and bilateral femoral dislocation were confirmed. Liver histology showed acute canalicular cholestasis with bile duct plugging. There was mild portal inflammation with ductular reaction. Collagen stain demonstrated portal and periportal fibrosis with focal portal–portal bridging (Fig. 2g). Some of the changes were thought to arise secondary to sepsis; however, the degree of fibrosis in particular was unusual and may relate to the patient’s underlying genetic condition. Histological analysis of the cerebellar cortex revealed patchy reduction/absence of Purkinje cells (Fig. 2k).

### Genome and RNA-seq identified a deep-intronic variant in *CDK5RAP3*

**Family A:** Chromosome microarray for AII-1 and AII-3 was unremarkable. No causative variants were identified with genetic diagnostic testing: Myotonic Dystrophy Type-1 testing (Victorian Clinical Genetics Services, VIC, Australia), *SMN1-*deletion testing (South Eastern Area Laboratory Services, NSW, Australia), Neuromuscular Panel (NSES v2; including arthrogryposis, congenital muscular dystrophy and myopathy genes; PathWest Laboratory, WA, Australia) and Epileptic Encephalopathy Panel (v2.2, 80 genes; Children’s Hospital at Westmead, NSW, Australia). A PCH Panel (Table S4, Children’s Hospital at Westmead, NSW, Australia) identified heterozygous variants of uncertain significance in *SEPSECS* (NM_016955.3:c1027-56_1027-53del) and *TSEN54* (NM_207346.2:c.767G > A), which were not pursued due to the absence of biallelic variants and likely autosomal recessive inheritance.

A family history indicative of a monogenic disorder prompted trio genome sequencing (AII-1, AI-1, AI-2) and RNA-seq (AII-1). No segregating, plausible variant(s) were identified in known Mendelian genes. The only plausible candidate was a homozygous deep-intronic variant, chr17(GRCh38):g.47974691G > A; *CDK5RAP3* NM_176096.3:c.334 + 243G > A (ClinVar Reference VCV004070917.1), predicted by SpliceAI to activate a cryptic donor splice-site.

**Fig. 3:**
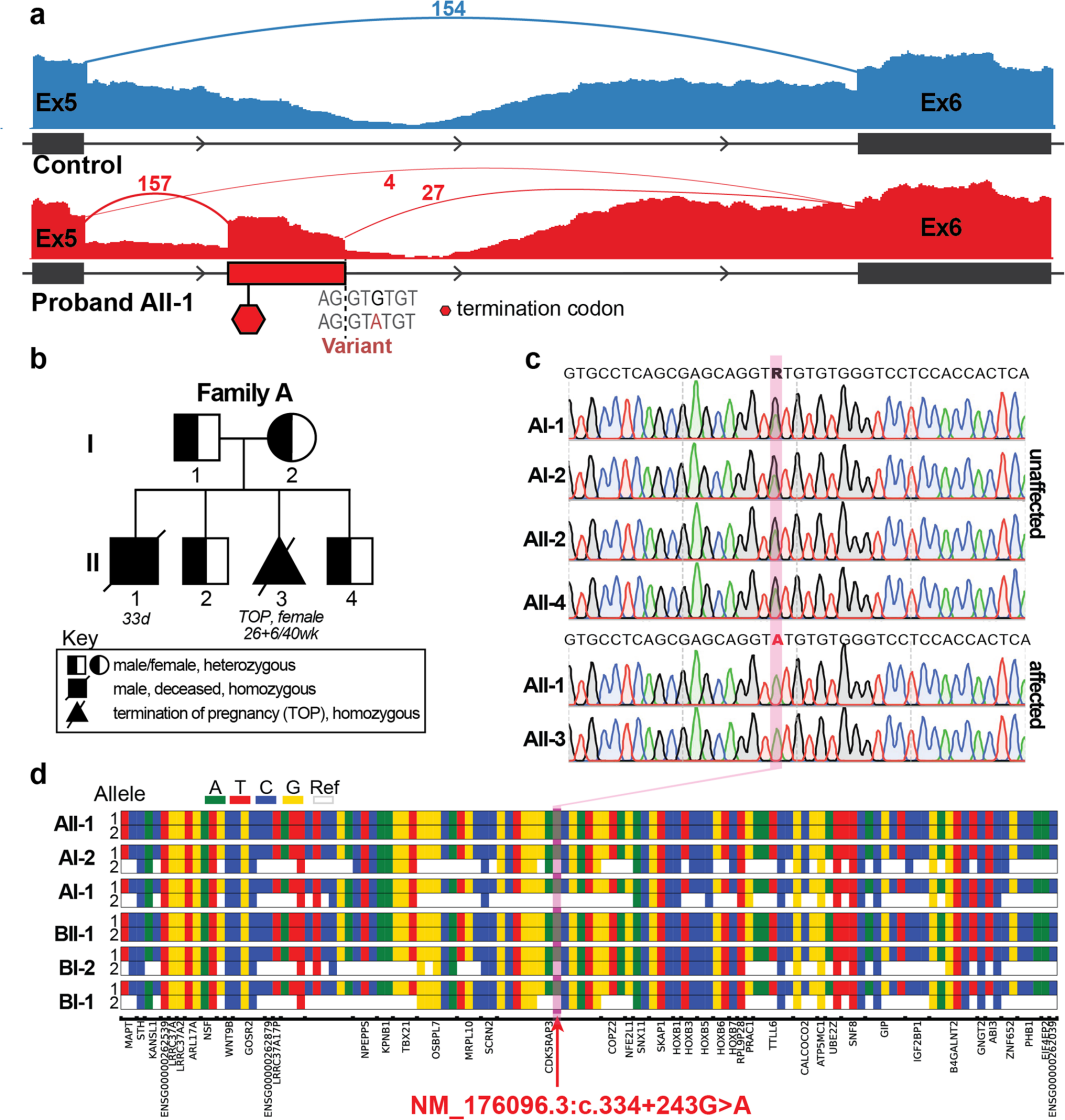
Segregating, biallelic deep-intronic *CDK5RAP3* variants cause aberrant splicing. **a** An RNA-seq of AII-1 (bottom/red, quadriceps muscle, age: 33 days) and a paediatric disease-control (top/blue: male, quadriceps muscle, age: 8 years) revealed aberrant inclusion of a deep-intronic PE, containing a PTC (red hexagon), between exons 4 and 5 in *CDK5RAP3* transcripts. Sashimi plot loops represent split reads; only 4 reads support canonical exon 4–5 splicing, while 157 reads support splicing from exon 5 to the PE acceptor site. The variant strengthens the PE donor (SpliceAI Donor gain *D*-score = 0.42) promoting mis-splicing. **b** Family A pedigree prepared in accordance with Ref. [5] and **c** Sanger sequencing traces showing segregation of the *CDK5RAP3* NM_176096.3:c.334 + 243G > A variant in homozygous affected siblings (AII-1, AII-3, filled symbols) and heterozygous, unaffected family members (AI-1, AI-2, AII-2, AII-4, half-filled symbols). Age-at-death is indicated for affected individuals. **d** Analysis of the shared haplotype harbouring the NM_176096.3:c.334 + 243G > A variant in both families. Colours represent the alternative nucleotide for 118 high-quality variants called in the region of homozygosity detected by exome in AII-1 (GRChg38 chr17:45,983,659–49,424,990) for each individual with exome data. Reference nucleotides are shown in white. Only variants with a genotype quality score > 35 for all individuals were included for analysis

RNA-seq (AII-1) confirmed *CDK5RAP3* c.334 + 243G > A caused PE/intronic sequence inclusion, encoding a premature termination codon (PTC, NP_788276.1;p.V112Gfs*8), predicted to trigger NMD (Fig. 3a). Minimal canonical splicing was detected, indicating the c.334 + 243G > A allele is hypomorphic (Fig. 3a). Low levels of residual *CDK5RAP3* splicing likely permitted survival of AII-1 until birth, in contrast to the embryonic lethality of mouse *Cdk5rap3* knockout [58, 69]. Pathogenicity of *CDK5RAP3* c.334 + 243G > A was further supported by the absence of individuals with homozygous loss-of-function variants in *CDK5RAP3* in gnomAD v4.1.0, by neurodevelopmental disorders linked to other UFMylation complex components, and by familial segregation (Fig. 3b, c).

**Family B**: SNP microarray showed no copy number change of significance. The Trio exome was non-diagnostic. Research reanalysis of the trio exome data identified the homozygous *CDK5RAP3* c.334 + 243G > A variant in BII-1. Both parents were heterozygous for this variant.

### Evidence for a shared founder allele in South Asian populations

Because the same variant was present in both families, we investigated the surrounding genetic region for a shared ancestral origin. Across 118 single nucleotide variants (SNVs) in both pedigrees (AII-1, AI-1, AI-2; BII-1, BI-1, BI-2), we identified a shared homozygous region of ~ 3.6–3.7 Mb (Fig. 3d), consistent with inheritance from a common ancestral haplotype and founder event many generations ago. Although both families are Nepalese, their differing ancestry, with only Family A reporting Indian heritage, argues against recent relatedness. Population data further support the likelihood of a South Asian founder allele: in gnomAD v4.1.0, eight of ten heterozygous individuals have South Asian ancestry.

### *CDK5RAP3* c.334 + 243G > A profoundly reduces canonical transcript and protein levels

**Fig. 4:**
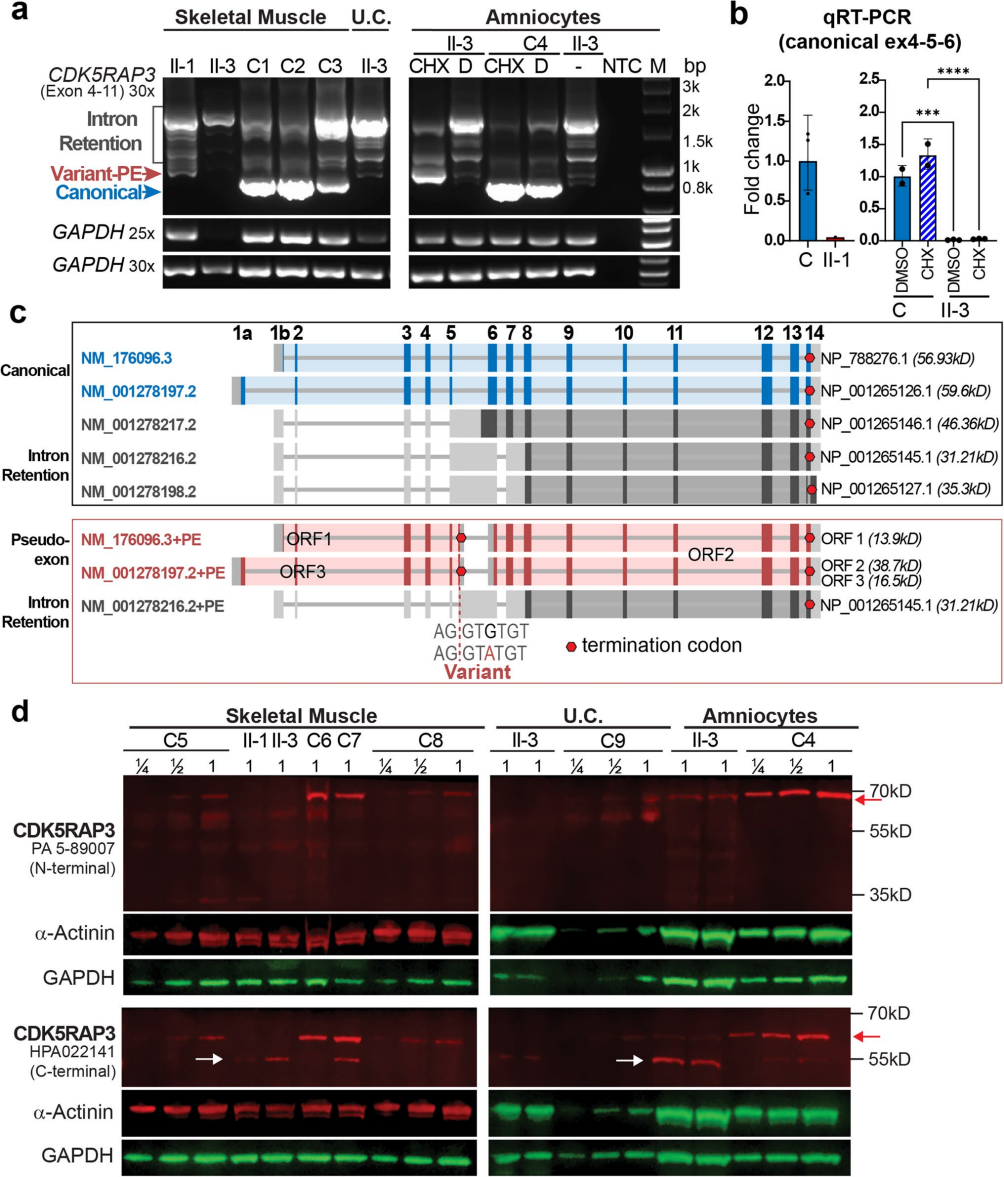
*CDK5RAP3* transcript and protein expression studies. **a** RT-PCR spanning *CDK5RAP3* exons 4–11. Two amplicons are detected in controls: canonically spliced (823 bp product, blue arrow; NM_176096.3 and NM_001278197.2 transcripts) and larger transcripts retaining intron 5 or introns 5 and 7 (“intron retention”, grey bracket; NM_001278217.2, NM_001278216.2 and NM_001278198.2). In AII-1 and AII-3, several *CDK5RAP3* transcripts retaining intronic sequences were present: (1) canonical transcript containing a 108 bp PE between exons 5 and 6 (“Variant-PE”, red arrow); (2) transcript containing 108 bp PE plus an additional 470 bp intronic sequence 3’ downstream and sometimes retaining intron 7 (grey bracket). **b** qPCR showing canonical splicing is reduced to 4.4% in AII-1 muscle and to 1.6% and 2.3% in AII-3 amniocytes treated with DMSO or CHX, respectively, compared to control. (***p = 0.0002, ****p < 0.0001 one-way ANOVA with Šídák’s multiple comparisons test). Treatment: CHX, DMSO (D) or untreated (–), NTC = negative template control, M = size marker, U.C. = umbilical cord. **c** Schematic of NCBI annotated *CDK5RAP3* transcripts (top) and aberrant transcript detected in AII-1 and AII-2 (bottom) with introns shown as lines and exons as boxes. The open reading frame (ORF)/coding sequence is shown in colour or dark grey, while 5’ and 3’ untranslated regions (UTR) are shown in light grey. Red hexagon = termination codon. **d** Western Blot using antibodies targeting a CDK5RAP3 N-terminal (PA5-89,007) or C-terminal (HPA022141) epitope. Full-length CDK5RAP3 (red arrow; predicted 57 kDa, observed 65 kDa) is detected with both antibodies. Additional bands (white arrow), consistent with alternative isoforms lacking the N-terminus, are detected only with the C-terminal antibody. Antibody validation confirmed PA5-89,007 has a low affinity for these proteins (Fig. S3). Ages: Proband: AII-1: 37 + 5/40 weeks’ gestation, AII-3: 26 + 6/40 weeks’ gestation; controls: C1: 4 m male, C2: 5y female, C3: 18/40 weeks’ unknown gender, C4: age/gender unknown, C5: 37/40, C6: 28/40, C7: 19/40, C8: 51 d, C9: 27/40 weeks’ gestation

RT-PCR spanning *CDK5RAP3* exons 4–11 was performed on mRNA from skeletal muscle (AII-1, AII-3), umbilical cord (AII-3), and amniocytes (AII-3). Note, no tissue was available for family B. Compared to controls, AII-1/ AII-3 specimens showed: i) undetectable canonical exon 4–11 splicing (full-length transcripts NM_176096.3 and NM_001278197.2; Fig. 4a, blue arrow); ii) inclusion of a 108-bp PE from intron 5 (red arrow in Fig. 4a and red transcripts in Fig. 4c); iii) aberrant splicing from exon 5 donor to the PE cryptic acceptor (chr17(GRCh38):47,974,688), retaining most of intron 5, with or without additional retention of introns 7 and/or 8 (Fig. 4a, c grey transcripts). CHX-treatment of AII-3 amniocytes increased PE-inclusion transcripts (Fig. 4a), consistent with NMD targeting PE-inclusion transcripts encoding a PTC.

*CDK5RAP3* shows complex alternative splicing, with transcripts retaining introns 5, 7, and/or 8 also present in controls (Fig. 4a–c grey transcripts). We examined the abundance of *CDK5RAP3* transcripts in GTEx Project V9 long-read transcriptomics data [[25]; Fig. S1; skeletal muscle, lung, liver, brain]. Notably, NM_001278217.2 expression was higher than the MANE Select (canonical) transcript NM_176096.3. Alternative transcripts NM_001278216.2 and NM_001278217.2 were particularly highly expressed in cerebellar hemispheres.

qRT-PCR confirmed that canonical exon 4–5–6 splicing was reduced in AII-1 skeletal muscle and AII-3 amniocytes to < 5% of control levels (Fig. 4b). Both siblings showed an overall reduction of all *CDK5RAP3* transcripts, suggesting that the apparent enrichment of intron 5-retaining transcripts in AII-1/AII-3 specimens observed by RT-PCR reflects increased relative amplification due to canonical isoform loss (Fig. S2).

Western blot using N- and C-terminal CDK5RAP3 antibodies (Fig. S3 for epitopes) confirmed that full-length CDK5RAP3 protein was reduced to < ¼ control levels in AII-3 amniocytes and below detectable levels in proband skeletal muscle and umbilical cord (Fig. 4d). A ~ 5–10 kD smaller protein product (white arrow, Fig. 4d) was detected by the C-terminal CDK5RAP3 antibody in proband skeletal muscle and amniocytes, potentially representing an alternate isoform translated from intron 5-retaining transcripts with an initiation methionine in exon 6 (Fig. 4b, grey transcripts). Western blot across developmental stages (13/40 weeks’ gestation to 31 years) suggests a ~ 5–10 kD smaller isoform may be physiologically present in skeletal muscle < 28/40 weeks’ gestation (Figs. 4d and S4).

### Putative retained CDK5RAP3 isoforms show poor UFL1-binding

**Fig. 5:**
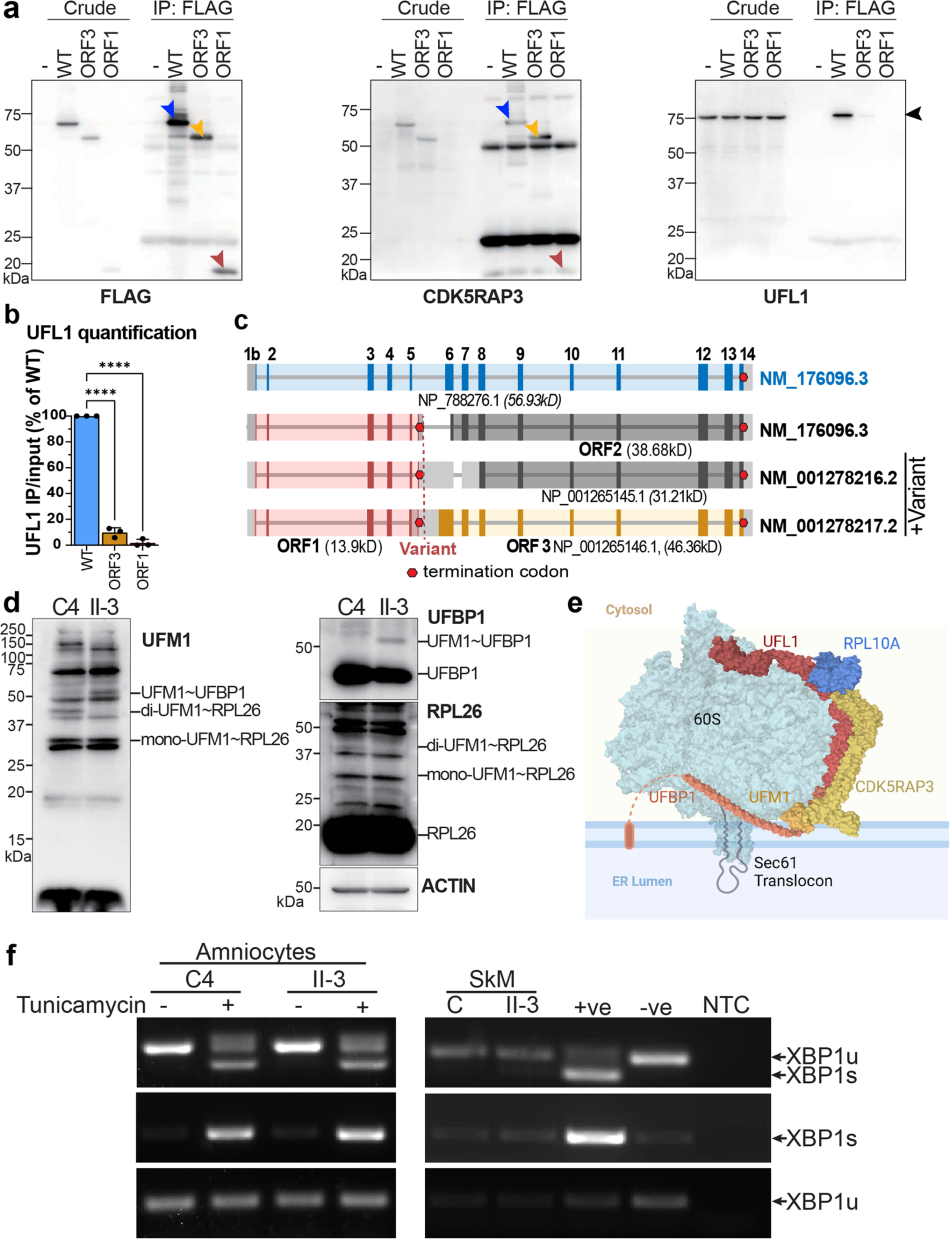
UFMylation and UPR assessment in CDK5RAP3 deficiency. **a** Co-IP to determine whether wt full-length CDK5RAP3, or shorter isoforms derived from ORF3 (NP_001265146.1) and ORF1, can complex with UFL1. FLAG-tagged CDK5RAP3 wt, ORF3 and ORF1 are detected with FLAG and CDK5RAP3 antibodies (arrows). Probing for UFL1 reveals only full-length wt CDK5RAP3 can co-precipitate UFL1, indicating interaction. In contrast, UFL1 co-precipitation was significantly reduced with ORF3 and ORF1 (9.8 ± 3.7% and 1.7 ± 2.8%, respectively; ***p < 0.0001 one-way ANOVA; quantification across 3 experiments in (**b**). Bar graphs show mean ± standard deviation. **c** Schematic illustrating proteins predicted to arise from wt and variant *CDK5RAP3* gene sequences. **d** Western blot of UFMylation pathway components (UFM1, UFBP1, RPL26) in AII-3 amniocytes showed increased UFMylation of UFBP1 and decreased di-UFPylation of RPL26, indicating dysregulation of the pathway. **e** Schematic illustrating complex formation of UFMylation proteins (UFBP1, UFM1, CDK5RAP3, UFL1) with the 60S ribosomal complex (including RPL10A and RPL26) at the ER membrane. Illustration was generated in Biorender and is based on crystal structures from RCSB PDB, 8QFC [44] and 6R7Q [59]. **f** RT-PCR analysis of *XBP1* non-canonical splicing using control (C4, C) and AII-3 skeletal muscle (SkM) or amniocytes treated with DMSO (–) or 2.5 μg/ml Tunicamycin ( +) for 5 h to induce the UPR. Primer information is in Table S2. *Top*: primers amplifying both spliced (XBP1s) and unspliced (XBP1u) products. *Middle*: forward primer bridging the excised region, amplifying only XBP1s. *Bottom*: forward primer within the excised region, amplifying only XBP1u. *+ve* positive control fibroblasts with UPR activated, *-ve*: negative control fibroblasts with no UPR activation, *NTC* no template control

CDK5RAP3 forms part of the UFM1 ribosome E3 ligase (UREL) complex, directly interacting with UFL1 to facilitate UFMylation of the 60S ribosomal subunit protein RPL26 [64]. This modification is essential for 60S subunit release from the SEC61 translocon (Fig. 5e) during ribosome stalling or normal termination of translation [44].

Although *CDK5RAP3* c.334 + 243G > A nearly abolished canonical protein (NP_788276.1) production, the 5–10 kD smaller observed protein product may represent the annotated alternative isoforms NP_001265146.1, unaffected by c.334 + 243G > A (Fig. 4c, d). NP_001265146.1 shares the C-terminal 395 amino acids with NP_788276.1 (Fig. S3), but has a short, distinct N-terminus (24 versus 111 amino acids).

Co-immunoprecipitation experiments compared the ability of wt, NP_001265146.1 (open reading frame-3; ORF3) and truncated mutant (ORF1) CDK5RAP3 isoforms to bind UFL1. Wt CDK5RAP3 bound UFL1 as expected [39], while NP_001265146.1 showed a markedly reduced UFL1 co-precipitation and ORF1 failed to pull down UFL1 (Fig. 5a–c).

### AII-3 amniocytes have a UFMylation defect

To determine whether CDK5RAP3 deficiency caused a UFMylation defect, we investigated known UFMylation substrates RPL26 and UFBP1 in AII-3 amniocytes. RPL26 is UFMylated on Lys132 and Lys134 [64, 65]. While mono-UFMylated RPL26 levels were unchanged, di-UFMylated RPL26 was reduced. UFMylated UFBP1 was enhanced in AII-3 amniocytes compared to control (Fig. 5d).

### Constitutive unfolded protein response (UPR) was not activated in skeletal muscle and amniocytes from affected individuals

Despite previous reports of ER stress in CDK5RAP3-deficient U2OS cells and hepatocytes [35, 69], we found no evidence of abnormal UPR activation in skeletal muscle or AII-3 amniocytes. *XBP1* splicing, a surrogate marker of ER unfolded protein induced stress and UPR induction [71], was not elevated at baseline in proband tissues (Fig. 5f). Upon tunicamycin treatment to elicit ER stress, *XBP1* splicing patterns were as expected and comparable between control C4 and AII-3 amniocytes (Fig. 5f), indicating preserved UPR responsiveness.

### Global proteomic profile alterations due to CDK5RAP3 deficiency

To assess the impact of the c.334 + 243G > A variant on the global proteome, we performed quantitative mass spectrometry comparing AII-3 and primary amniocytes derived from healthy control C4 (‘Proteomics Dataset 1’, Table S5, 4 replicas each). We identified 6826 unchanged, 188 significantly upregulated and 288 significantly downregulated proteins (Fig. S5**a**) with CDK5RAP3 among the significantly reduced proteins (*log2FC*: -1.9; 26.8% of control, q < 0.00001) consistent with Western blot results (Fig. 4d).

Given CDK5RAP3 is a UFMylation substrate adaptor, we examined UFMylation pathway components (Fig. S5b, Table S6). KIF11 (aka EG5), a recently identified UFMylation substrate [40], was significantly upregulated (*log2FC*: *1.4,* q < 0.001). Other substrates EIF6 [60], SLC7A11 [68], P4HB [81] and UFL1 showed modest changes below the significance threshold (> 0.50; *log2FC* of 0.34, 0.36, -0.38 and -0.28, respectively; *q* < 0.0001). Confirmatory Western blot did not demonstrate a reduction in UFL1 levels in AII-1 and AII-3 (Fig. S6).

Pathway enrichment analysis using Metascape [80] revealed the GO cellular components (CC) “ECM” to be the most significantly downregulated summary term, represented by specific pathway terms such as “ECM organisation”, “Non-integrin membrane-ECM interactions”, and “Assembly of collagen fibrils and other multimeric structures” (Fig. S5c; Table S7).

Interestingly, ECM, via different subsets of ECM proteins, also appeared among upregulated pathways, represented by GO CC “External encapsulating structures”, GO Molecular Functions (MF) “S100 protein binding”. Several cell cycle and mitotic progression pathways and cellular components pathways were also significantly upregulated (e.g. GO biological processes (BP) “Mitotic cell cycle”, reactome gene sets “Mitotic prometaphase” and “condensation of prometaphase chromosomes” together with “Golgi-to-ER retrograde transport”, which share overlapping protein components [e.g. KIF11, KIF4A/B, and KIF21A]; Fig. S5d). Additional themes included cytoskeletal organisation and cell attachment, heat stress, and growth factor signalling. Overall, proteome findings highlight global ECM and cell attachment dysregulation in AII-3 amniocytes. Themes align with prior studies linking CDK5RAP3 to cell cycle regulation, cell growth and cancer-related processes such as metastasis and growth factor signalling [41, 42, 49, 63, 67, 78] as well as the role of UFMylation in microtubule network and centrosome organisation [35].

### ASO restoration of CDK5RAP3 protein confirms modulation of ECM organisation, cell adhesion and migration

**Fig. 6:**
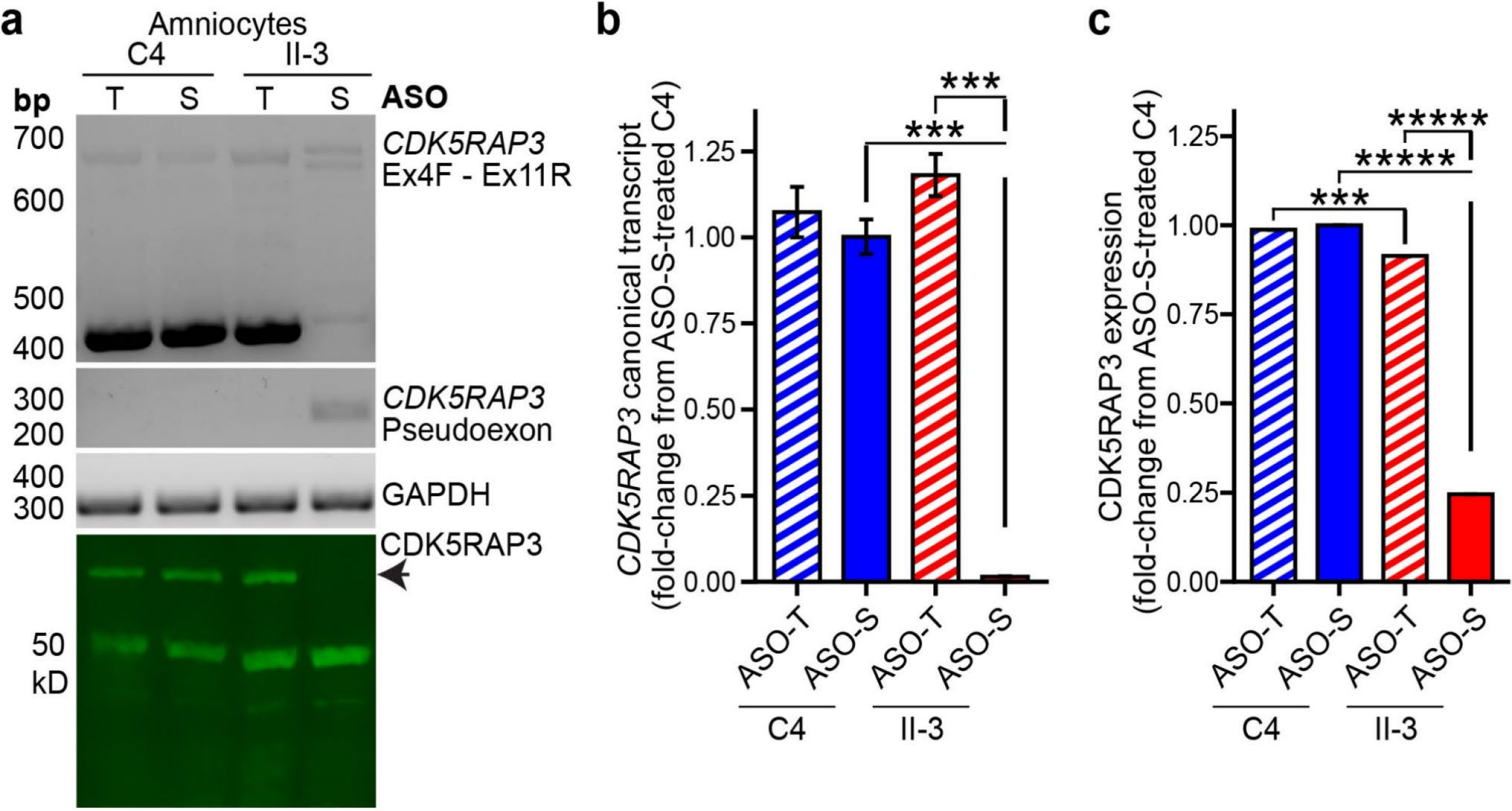
An ASO targeting the variant PE donor restored full-length *CDK5RAP3* transcript and protein expression. Transcript/protein impact of *CDK5RAP3* PE donor targeting ASO (ASO-T) and scrambled control ASO (ASO-S) nucleofected into AII-3 and C4 primary amniocytes was assessed with **a** RT-PCR and Western blot (C-terminal antibody HPA022141). ASO-T restored canonical *CDK5RAP3* splicing, and rescued full-length CDK5RAP3 protein expression in AII-3 amniocytes. ASO-S had no effect. **b** Quantitative RT-PCR (***p < 0.0001, pairwise t-tests between samples) and **c** mass spectrometry (***q < 0.0001, *****q < 0.00001) confirmed restoration of *CDK5RAP3* transcript levels and protein levels, reaching 118.0% and 91.4% of control levels, respectively

To mitigate caveats from studying one affected cell line, we developed a complementation assay to identify pathways specifically disrupted by CDK5RAP3 deficiency and restored upon re-expression. A targeted antisense oligonucleotide (ASO-T) was designed to block the cryptic donor site activated by c.334 + 243G > A. ASO-T or a scrambled control ASO (ASO-S) was introduced via nucleofection into control C4 and AII-3 amniocytes, and harvested after 72 h. ASO-T-mediated restoration of canonically spliced *CDK5RAP3* transcripts and protein to control levels was confirmed by RT-PCR, qRT-PCR, Western blot, and mass spectrometry (Fig. 6).

Proteomic/phosphoproteomic principal component analysis (PCA) showed overlapping ASO-T and ASO-S clusters for control cells, indicating minimal off-target effects (Fig. S7; Proteomics Dataset 2’ and ‘Phosphoproteomics Dataset’ in Table S8–S9). In contrast, proband ASO-T and ASO-S clusters were distinct, with ASO-T-treatment shifting profiles towards the control along PC1.

**Fig. 7:**
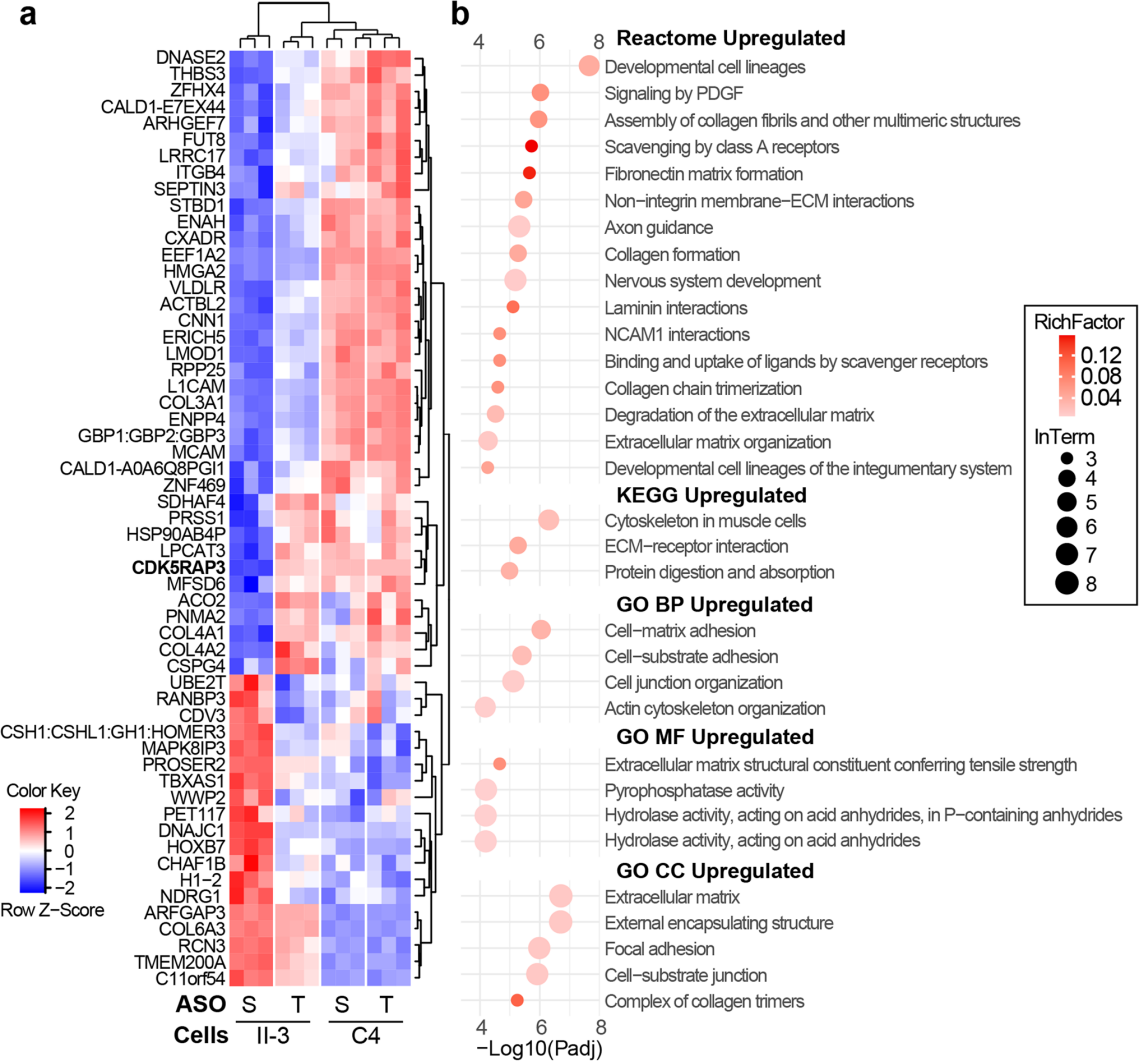
Proteomic analysis of ASO-treated amniocytes highlighting CDK5RAP3-sensitive pathways. **a** Heat map of standardised levels (z-scores) of differentially abundant proteins that were sensitive to rescue by ASO-T-mediated restoration of CDK5RAP3 protein (‘ASO-T Rescue Set’). **b** Dot plot of significantly enriched “member term” pathways identified by Metascape analysis of the Rescue Set. Pathway enrichment is quantified by “RichFactor” (ratio of restored proteins to total proteins in the pathway) and “InTerm” (number of restored proteins within each pathway). No pathways were significantly downregulated by ASO-T. Note, the direction of the change reflects the comparison between ASO-T and ASO-S-treated AII-3 cells. Therefore, pathways shown as upregulated in this figure are downregulated in the C4 vs AII-3 comparison

**Fig. 8:**
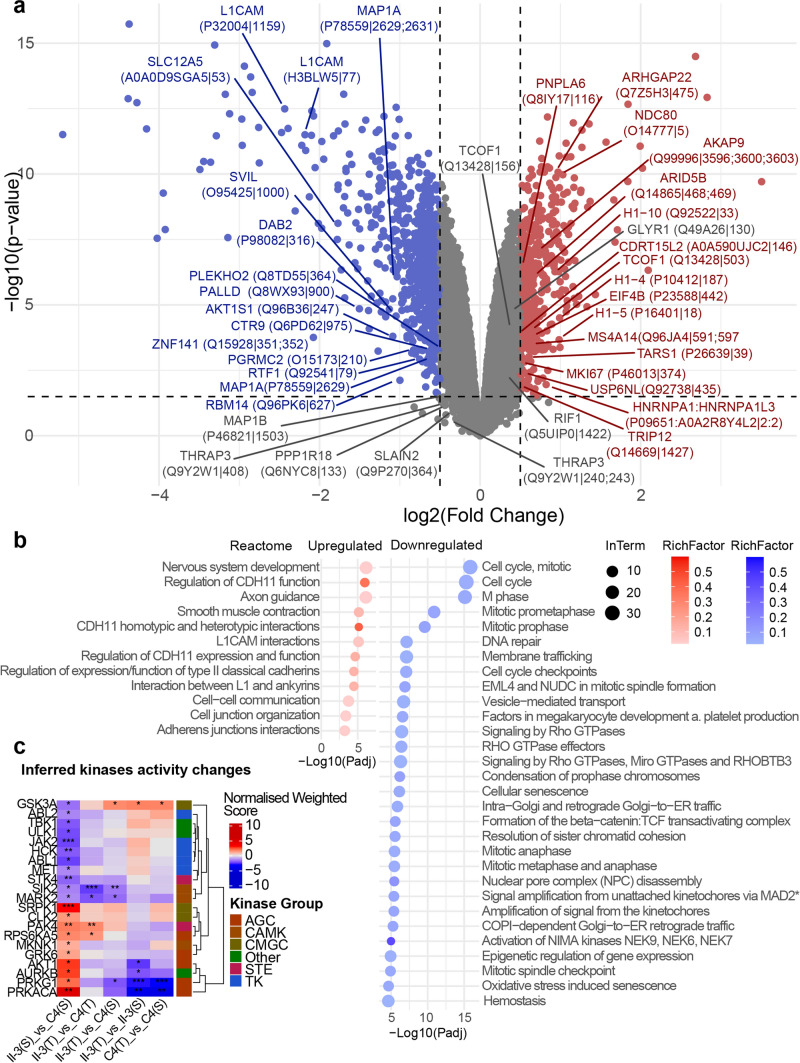
Phosphoproteomic analysis of ASO-treated amniocytes reveals CDK5RAP3-sensitive pathways. **a** Volcano plot of differentially abundant phosphorylation sites between AII-3 and C4 ASO-S-treated amniocytes. Labels indicate the 20 most upregulated (red) and downregulated (blue) phosphorylation sites among the ASO-mediated CDK5RAP3 restoration ‘Rescue Set’ proteins. Grey points represent non-significant changes. **b** Dot plot of Reactome pathways significantly enriched among phosphosites restored by ASO-T. “RichFactor” represents the ratio of restored proteins to total proteins in each pathway. “InTerm” indicates the number of restored proteins per pathway. *Full GO term: Amplification of signal from unattached kinetochores via a MAD2 inhibitory signal (truncated on figure for space consideration). Directionality of change reflects the comparison between AII-3 treated with ASO-T vs ASO-S; thus, pathways shown as upregulated here are downregulated in the C4 vs AII-3 comparison. **c** Heat map showing kinases with significant changes in inferred kinase activity between ASO-S-treated AII-3 and C4 amniocytes (*p < 0.05, **p < 0.005, ***p < 0.001). (S): ASO-S, (T): ASO-T

We identified an “ASO-T Rescue Set” of 57 proteins and 505 phosphopeptides whose expression was partially or fully restored towards control levels after 72 h of ASO-T treatment in proband cells (Figs. 7a and 8a; Table S8–S9). Pathway analysis using the ‘ASO-T Rescue Set’ resulted in the detection of only 32 up- and no downregulated reactome/KEGG/GO pathways (Table S10, Fig. 7b). Identified pathways related to ECM, cell adhesion (cell–matrix, cell–substrate, ECM–receptor interactions), cytoskeletal networks, neurodevelopmental signalling (e.g. PDGF signalling, axon guidance, and neuronal migration) as well as scavenger receptor-mediated uptake, NCAM1 interactions, and protein digestion and absorption.

A subset of rescued proteins (26 of 57) did not cluster within curated pathway terms, but several have known roles consistent with CDK5RAP3-linked processes and pathways detected in enrichment analysis. For example, NDRG1 and MAPK8IP3 have been linked to neurological disorders [31, 79] with MAPK8IP3 functionally linked to axonal transport [76]. DNAJC1, an ER and plasma membrane localised co-factor of the molecular chaperone HSPA5, plays roles in cancer, ECM reorganisation, proliferation and migration [23, 75]. CHAF1B and H1-2 (HIST1H1C) are important for chromatin structure and organisation.

Pathway enrichment analysis of the phosphoproteomic ‘ASO-T Rescue Set’ revealed enrichment of 215 upregulated and 236 downregulated reactome/KEGG/GO pathways (Table S11, Fig. 8b, Fig. S9). ASO-T selectively restored downregulated phosphorylation sites involved in actin filament remodelling required for motility and shape changes at the cell leading edge (actin binding, actin filament bundle, lamellipodium, cell cortex, regulation of cytoskeleton organisation), cell morphogenesis and polarity (including regulation of protein localisation to cell periphery), cell–cell/cell–matrix adhesion and endocytosis. Conversely, phosphorylation sites previously upregulated and restored with ASO-T but not ASO-S centred on nuclear organisation, DNA metabolism, and cell cycle regulation. Prominent themes included genome architecture, mitosis/meiosis chromatin/chromosome dynamics and microtubule-organising centre for spindle formation and chromosome segregation (summary terms such as chromosome organisation/segregation, mitotic prophase, heterochromatin, chromatin binding). Additionally, nucleic acids and transcription processes (e.g. DNA metabolic process, DNA-templated transcription, and positive regulation of RNA polymerase I-mediated transcription and ribonucleoprotein granule) and processes of dynamic nuclear architecture regulation and nuclear transport (e.g. nuclear matrix, nuclear pore complex [NPC] disassembly, and regulation of protein-containing complex assembly) were also represented. Finally, terms such as membrane trafficking, cadherin binding, and macromolecular conformation isomerase activity suggest integration of nuclear/cell cycle processes with protein folding and cell–cell adhesion.

Except for CDK5RAP3, no UFMylation components were present in the ASO-T Rescue Set suggesting that the major CDK5RAP3-dependent changes occur downstream of the ligase complex and do not affect UFMylation pathway protein abundance (Table S12).

However, phosphoproteomic profiling revealed CDK5RAP3-dependent regulation of two key proteins: UFL1 and SLC7A11 (Table S13).

UFL1 S462 was hypophosphorylated in ASO-S-treated AII-3 amniocytes compared to control [log2FC -0.58]. UFL1 S462 phosphorylation promotes UFL1 recruitment and activation following DNA damage by enhancing its E3 ligase activity. The kinase responsible, ATM (ataxia-telangiectasia mutated) [57], is central to the DNA-damage response and also plays key roles in cerebellar development, neuronal migration and maturation [reviewed in 6]. ASO-T-treatment restored near-normal UFL1 S462 phosphorylation levels, implicating CDK5RAP3 and ATM as upstream regulators of UFL1 activity. Other UFL1 phosphorylation sites (S431, S458, S789, S792) were detected but did not meet differential phosphorylation significance thresholds. Conversely, ASO-T reversed hyperphosphorylation of cystine-glutamate antiporter xCT/SLC7A11 at S26 (log2FC 0.5), a modification known to inhibit transporter activity via mTORC2–AKT signalling [26]. SLC7A11 is stabilised by UFMylation, acting as a key regulator of ferroptosis and mediator of the cancer treatment metformin [68]. While no direct link between CDK5RAP3 and SLC7A11 has been established, our finding suggests CDK5RAP3 may influence SLC7A11 regulating signalling pathways. Inferred kinase activity analysis revealed elevated activity of MKNK1, GRK6, AKT1, AURKB, PRKG1 and PRKACA, which were partially or fully restored to near/normal levels with ASO-T-treatment (Fig. 8c).

## Discussion

We present the first evidence linking biallelic variants in *CDK5RAP3*, encoding a key UFMylation adaptor, to a neonatal‑lethal neurodevelopmental disorder characterised by foetal akinesia, PCH, arthrogryposis, and liver fibrosis. *CDK5RAP3* c.334 + 243G > A appears to represent a South Asian founder variant, with important implications for pre‑conception carrier screening and for families experiencing recurrent foetal or early infantile death in these communities. Despite extensive efforts, including Matchmaker Exchange queries and outreach to global collaborators, no further families were identified, suggesting *CDK5RAP3* c.334 + 243G > A is either exceptionally rare or may often go undetected due to neonatal lethality and its deep-intronic location, which makes detection and interpretation difficult.

Biallelic *Cdk5rap3* knockout causes embryonic lethality in mice [69]. Thus, biallelic loss-of-function variants in *CDK5RAP3* may be incompatible with human development as well, and only hypomorphic variant(s) with residual full-length CDK5RAP3 expression, such as c.334 + 243G > A, may be identified in individuals who survive to birth.

Notably, c.334 + 243G > A does not affect certain alternative transcripts predicted to encode a C-terminal ORF. GTEx long-read RNA-Seq data indicate these isoforms have expression particularly in the cerebellum. We provide preliminary evidence supporting potential translation of alternative *CDK5RAP3* transcripts: Western blot detects a band ~ 5–10 kDa smaller than full-length CDK5RAP3 in tissues and cells from AII-1/AII-3 and in human developmental skeletal muscle (< 28/40 weeks’ gestation). Definitive identification of the Western blot bands is still required to confirm the presence and functional relevance of alternative CDK5RAP3 isoforms. If present, these shorter C-terminal isoforms may retain partial function; however, immunoprecipitation data indicate that their markedly reduced UFL1-binding capacity limits their role in UFMylation, suggesting that only full-length CDK5RAP3 serves as an efficient adaptor.

Consistent with impaired UFMylation activity, AII-3 amniocytes showed decreased RPL26 di-UFMylation, consistent with CDK5RAP3’s role in facilitating conjugation of the second UFM1-moiety (Walczak et al., 2019). RPL26 di-UFMylation is proposed to regulate dissociation of the 60S ribosomal subunit from the SEC61 translocon during translation termination or stalling, as part of ER-associated ribosome quality control (Makhlouf et al., 2024). We also observed UFBP1 hyper-UFMylation, consistent with knockout models and hypothesised to result from increased accessibility of Lys267 to UFL1 in the absence of CDK5RAP3 [29]. Despite prior reports of ER stress in *Cdk5rap3*-deficient cells [35, 69], we found no evidence of constitutive UPR activation in proband tissues or cells. *XBP1* splicing was not elevated compared to controls at baseline or following tunicamycin treatment. This may reflect the hypomorphic *CDK5RAP3* allele and/or compensatory activity of alternate CDK5RAP3 isoforms, sufficient to maintain ER homeostasis under baseline cell culture conditions.

CDK5RAP3 interacts with cyclin-dependent kinase 5 (CDK5) [12], a protein essential for neuronal migration, cortical development, and cytoskeletal organisation [54]. Biallelic loss-of-function *CDK5* variants cause lissencephaly-7 with cerebellar hypoplasia [43, 50]. Furthermore, several UFMylation pathway genes are associated with severe neurodevelopmental syndromes and *Cdk5rap3* knockout in mice is lethal [10, 58, 70], supporting pathogenicity of CDK5RAP3 deficiency. While individuals with CDK5RAP3 deficiency share neurodevelopmental and growth restriction features overlapping those of *CDK5-* and UFMylation-related disorders, they also show liver and skeletal muscle involvement. It is currently unclear whether skeletal muscle involvement represents a primary effect of CDK5RAP3 deficiency on muscle. While observed skeletal muscle histopathology does not resemble a typical denervation pattern, we cannot rule out anomalies secondary to innervation defects related to the neuromuscular junction or to spinal cord anterior horn cell dysfunction warranting further investigation. However, liver involvement is consistent with findings in *Cdk5rap3* knockout mice, which exhibit defects in neurogenesis and hematopoiesis, alongside impaired liver development/regeneration, and hepatic inflammation [10, 11, 70]. Multi-system involvement may reflect integrated roles of CDK5RAP3 in UFMylation regulation and genome maintenance, leading to additional vulnerabilities in liver and muscle.

We developed a targeted antisense oligonucleotide that effectively restored canonical *CDK5RAP3* splicing and full-length protein expression in patient-derived cells. This rescue allowed us to directly assess which molecular abnormalities were reversible and to identify CDK5RAP3-dependent pathways. Dysregulated processes included ECM organisation, cell adhesion, cytoskeletal networks, mitosis, nuclear architecture, and neurodevelopmental signalling, aligning with the observed neuronal migration defects and supporting prior studies implicating CDK5RAP3 in cell cycle regulation, metastasis, and growth factor signalling. A striking phosphoproteomic finding implicates CDK5RAP3 as an upstream regulator of UFL1 phosphorylation at S462, a site known to be regulated by ATM signalling [57]. ATM, beyond its canonical role in DNA-damage response, has been linked to a human genetic disorder (Ataxia-telangiectasia) and is increasingly recognised for its involvement in cerebellar development, neuronal migration, and maturation [17, 73], processes that align closely with the histopathological abnormalities observed in the affected individuals described here. Another known UFL1 phosphorylation site, T426 (phosphorylated by AKT and involved in ArpC4 UFMylation [77]), was not detected within our dataset.

## Conclusions and limitations

Our phenotypic, molecular, and functional data provide strong evidence that CDK5RAP3 deficiency leads to a severe neurodevelopmental disorder and may compromise muscle and liver function in humans. The proteomic and phosphoproteomic datasets indicate that CDK5RAP3 exerts broader cellular functions beyond UFMylation. However, the consistent, biologically plausible pathway disruptions we detected require orthogonal confirmatory experiments to allow deeper mechanistic insights. Our experimental findings remain constrained by the availability of only a single patient-derived amniocyte line. Additionally, amniocytes may not fully recapitulate CDK5RAP3 neuron-specific effects and future, larger studies using human neuronal model systems will be critical to dissect the neurodevelopmental consequences of CDK5RAP3 deficiency.

While ASO therapy is unlikely to reverse the neurodevelopmental phenotype, our ASO studies were valuable to confirm that c.334 + 243G > A drives the complex splicing defects and to provide a complementation control that strengthened our mechanistic and proteomic conclusions. Moreover, the ability to temporally restore full-length canonical CDK5RAP3 offers a unique opportunity to interrogate UFMylation and phospho-signalling pathways in neuronal organoids. The ASO rescue adds to growing evidence that deep-intronic variants are amenable to ASO-mediated splice-correction approaches [2, 19, 34]. Together, our findings support CDK5RAP3 as a central regulator at the intersection of UFMylation, neuronal migration, cellular signalling and stress response pathways.

## Supplementary Information

Below is the link to the electronic supplementary material.

Supplementary file1 (PDF 4587 KB)

Supplementary file2 (XLSX 2781 KB)

Supplementary file3 (XLSX 110 KB)

Supplementary file4 (XLSX 9930 KB)

Supplementary file5 (XLSX 21002 KB)

Supplementary file6 (XLSX 43 KB)

Supplementary file7 (XLSX 93 KB)

## Data Availability

All data supporting the findings of this study are available within the article and its Supplementary Information files. Raw genomic, transcriptomic, and proteomic datasets were not made publicly available due to ethical and consent restrictions but are available from the corresponding author upon reasonable request.
