# Supplementary material for: A severe neurodevelopmental syndrome linked to a South Asian founder variant in the UFMylation adaptor CDK5RAP3
Source: Acta Neuropathol. 2026 Apr 27;151(1):48. doi: 10.1007/s00401-026-03017-2 (PMC13121207; doi:10.1007/s00401-026-03017-2)
Supplement: Supplementary file 1 — Supplementary file1 (PDF 4587 KB) [file 401_2026_3017_MOESM1_ESM.pdf]

A Severe Neurodevelopmental Syndrome Linked to a South Asian Founder Variant in the UFMylation Adaptor *CDK5RAP3*.

Michaela Yuen[1,2,3,t], Katharine Zhang[1,2,3,t], Rhett G Marchant[1,2,3], Ryosuke Ishimura[4], Mark Graham[5,6], May Aung-Htut[7,8], Samantha Bryen[9,10], Rocio Rius[9,10,11], Lee Marshall[12], Nader Aryamanesh[5,12], Gregory Dziaduch[1,2,3], Himanshu Joshi[1,2,3], Ben Weisburd[13], Steve D Wilton[7,8], Meredith Wilson[3,14], Russell Gear[15,16], Lucy Hennington[17,18], Stephanie Lau[19], Helen Doyle[20], Michael Krivanek[20], Richard J Leventer[15,21,22], Susan M White[11,16,23], Sarah A Sandaradura[1,3,14], Masaaki Komatsu[4], Frances J Evesson[1,2,3*], Sandra T Cooper[1,2,3*]

[t,*] These authors contributed equally to this work.

1. Kids Neuroscience Centre, Kids Research, The Children's Hospital at Westmead, Westmead, NSW 2145, Australia
2. Functional Neuromics, Children's Medical Research Institute, Westmead, NSW 2145, Australia
3. Neuroscience, School of Medical Sciences, Faculty of Medicine and Health, The University of Sydney, Camperdown, NSW 2006, Australia
4. Department of Physiology, Juntendo University Graduate School of Medicine, Tokyo 113-8421, Japan
5. Children's Medical Research Institute, Faculty of Medicine and Health, The University of Sydney, Camperdown, NSW 2006, Australia
6. Biomedical Proteomics, Children's Medical Research Institute, Westmead, NSW 2145, Australia
7. Personalised Medicine Centre, Health Futures Institute, Murdoch University, Murdoch, WA 6150, Australia.
8. Perron Institute for Neurological and Translational Science, Perth, WA 6009, Australia.
9. Centre for Population Genomics, Garvan Institute of Medical Research, UNSW Sydney, Darlinghurst, NSW 2010, Australia
10. Centre for Population Genomics, Murdoch Children's Research Institute, Parkville, VIC 3052, Australia
11. Department of Paediatrics, University of Melbourne, Parkville, VIC 3010, Australia.
12. Bioinformatics Group, Children's Medical Research Institute, Westmead, NSW 2145, Australia
13. Broad Institute at MIT and Harvard, Boston, MA 02142, USA
14. Department of Clinical Genetics, The Children's Hospital at Westmead, Westmead, NSW 2145, Australia

15. Murdoch Children's Research Institute, Parkville, VIC 3052, Australia
16. Clinical Genetics Department, The Mercy Hospital for Women, Heidelberg, VIC 3084, Australia
17. Mercy Perinatal, Mercy Hospital for Women & Austin Health, Heidelberg, VIC 3084, Australia
18. Alfred Health, Melbourne, VIC 3004, Australia
19. Anatomical Pathology Department, Austin Health, Melbourne, Heidelberg, VIC 3084, Australia
20. Department of Histopathology, The Children's Hospital at Westmead, Westmead, NSW 2145, Australia
21. Department of Neurology, The Royal Children's Hospital Melbourne, Parkville, VIC 3052, Australia
22. Department of Paediatrics, University of Melbourne, Melbourne, VIC 3010, Australia.
23. Victorian Clinical Genetics Services, Murdoch Children's Research Institute, Parkville, VIC 3052, Australia

Correspondence to: Michaela Yuen

The Children's Medical Research Institute, 214 Hawkesbury Road Westmead NSW 2145

myuen@cmri.org.au; +61 2 9845 1455

Supplementary Methods

Proteome and Phosphoproteome data analysis

Raw data processing and normalisation: Data searching, cleaning and normalisation was performed as described [37]. MaxQuant v2.6.3.0 [12] was used for searching, including phosphorylation (on Ser/Thr/Tyr) as a variable modification. The *Homo sapiens* reference proteome (downloaded on 24/02/2025) was used for analysis and included canonical and isoform sequences with 83,385 entries and 20,644 genes. Protein reporter intensity corrected values and peptide reporter intensity corrected values from MaxQuant were imported into R (v4.3.3) for downstream analysis for proteome and phosphoproteome, respectively.

For proteome analysis, proteins identified by only modified peptides, reverse peptides or potential contaminant peptides were removed. Proteins were filtered for any row containing >0 missing values. If duplicated gene symbols existed, a single UniprotKB protein accession was kept based on the highest protein evidence, reviewed status, canonical status and then the lowest isoform number. For phosphoproteome analysis, peptides with no phosphosites, reverse peptides or potential contaminant peptides and peptides with phosphosite probabilities <0.75 were discarded from analysis. For multimapped phosphopeptides, a single UniprotKB protein accession was kept based on the best protein. Peptides with the same UniprotKB protein accessions and phosphosite locations were summed to phosphosites for further analysis. Phosphosites were filtered for any row containing >0 missing values.

Proteomic and phosphoproteomics intensities were log2-transformed and Variance Stabilizing Normalisation (VSN) applied using the vsn R package (v3.74.0) [22]. An ANOVA model was used to identify 500 negative control genes (adjusted p-value $\geq$ 0.5 and $\geq$ 0.25 for proteome and phosphoproteome, respectively). These genes were used for RUVIII unwanted variation removal, adjusting for desired component number (k = 4, k = 2, k = 4 for ‘Proteomics Dataset 1’, ‘Proteomics Dataset 2’ and Phosphoproteomics, respectively) using the ruv package (v0.9.7.1) [18, 28]. Following data cleaning, among the accepted proteins (7110), 10.52% (748) had 1 unique peptide and 89.48% (6,362) had more than 2 unique peptides.

Differential protein and phospho-site abundance analysis: Differential protein and phospho-site abundance was assessed using the limma R package. The log2-transformed proteomic intensities were fitted to a linear model, with empirical Bayes moderation to shrink variance estimates across proteins. Adjusted p-values were computed using the Benjamini–Hochberg procedure to control the false discovery rate (FDR) [4, 35]. Significant differentially expressed proteins were defined as those with q-values < 0.05 and absolute log2-fold changes ($|log2FC|$) > 0.5.

Pathway analysis: Pathway enrichment analysis was performed using Metascape (http://metascape.org/)[42]. Multimapped peptides within gene sets were separated into individual proteins. Enriched Gene Ontology (GO) terms (Biological Processes, Molecular Function, Cellular Compartment), KEGG Pathway, and Reactome pathways were identified using default analysis settings (Min overlap: 3, p-value cutoff: 0.01, Min enrichment: 1.5), with background genes set to all *H. sapiens* genes. The output was manually curated to discount pathways supported by <3 uniquely mapped proteins and FDR > 0.05 from the inbuilt Benjamini-Hochberg adjustment. Pathways were ranked by p-value, and up to 20 top "summary" and 30 top "member" terms were visualised.

Phosphoproteomic Kinase Activity Analysis: Differentially expressed phospho-sites linear model results was used to identify changes in kinase activity using the KinSwingR package (v1.20.0). PhosphoSitePlus database was used to identify kinase substrates, while the UniProtKB database allowed for the classification of each kinase into their respective kinase family group. Significant kinase activity changes were defined as those with probability score < 0.05.

Supplementary Figures and Tables

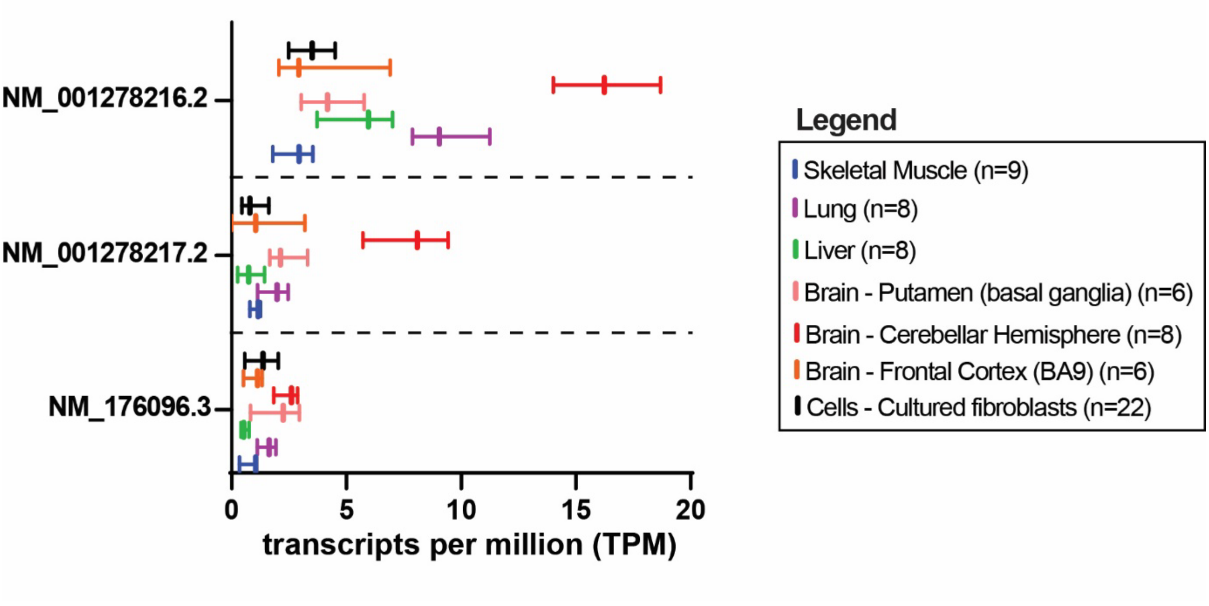

Fig. S1 *CDK5RAP3* transcript expression – long read data

The Genotype-Tissue Expression (GTEx) Project V9 long read transcriptomics data [19] was used to interrogate tissue specific expression of three *CDK5RAP3* transcripts, canonical (NM_176096.3) and two alternative transcripts (NM_001278216.2, NM_001278216.2), in a select set of tissues. Alternative transcripts are particularly highly expressed in the cerebellar hemisphere and lung. The data used for this analysis was obtained from the GTEx Portal (phs000424.v9) on 30th May 2022. The Genotype-Tissue Expression (GTEx) Project was supported by the Common Fund of the Office of the Director of the National Institutes of Health, and by NCI, NHGRI, NHLBI, NIDA, NIMH, and NINDS.

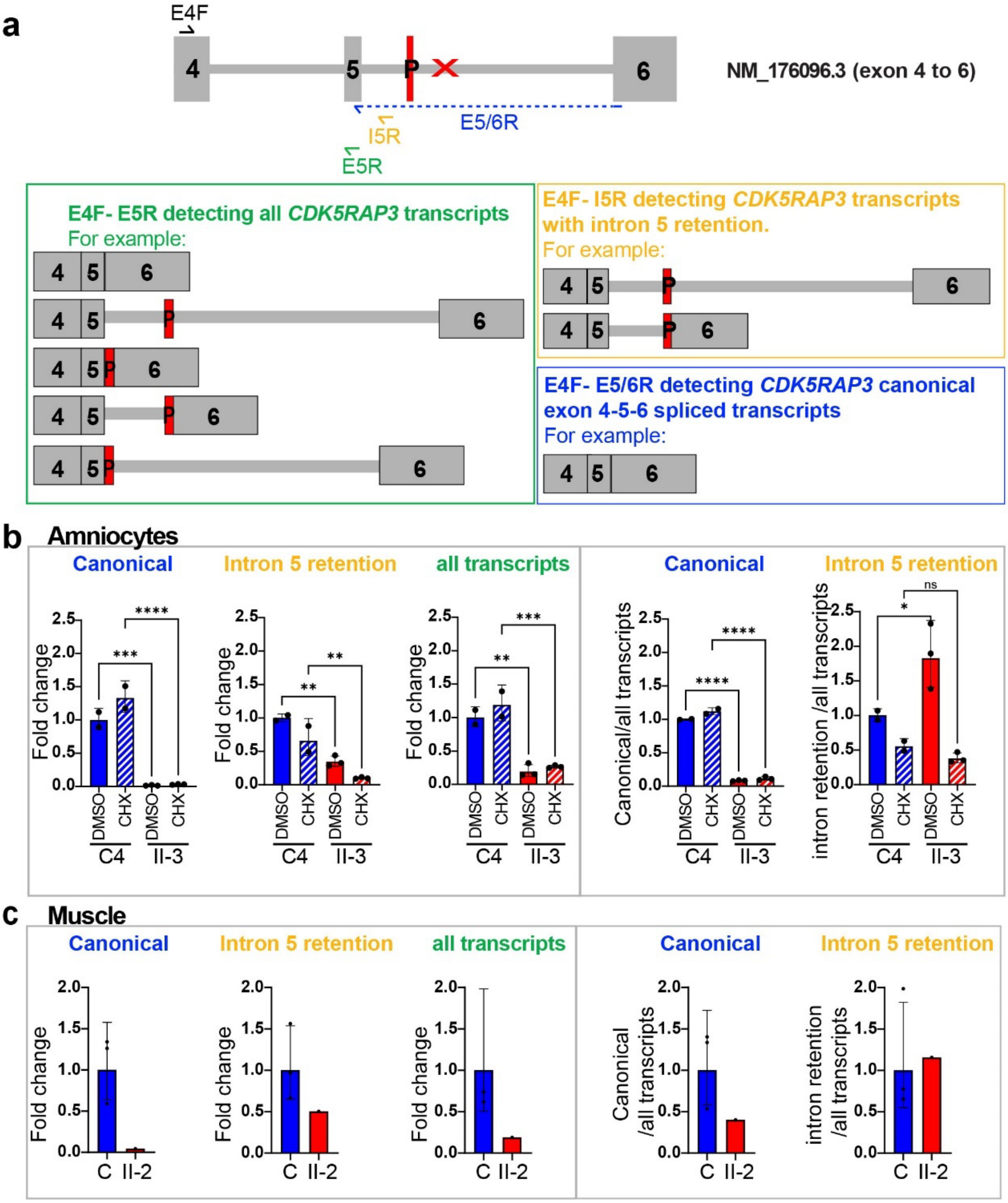

Fig. S2 qPCR confirms a reduction of all *CDK5RAP3* transcripts

(**a**) Schematic illustrating qPCR assays specifically targeting various *CDK5RAP3* transcripts: Green box – all transcripts (forward and reverse primer in exon 4 and 5, respectively), yellow box – intron 5 retention transcripts (forward and reverse primer in exon 4 and intron 5, respectively), and blue box - canonically spliced transcripts (forward and reverse primer in exon 4 and at exon 5/6 junction, respectively). qPCR results of (**b**) amniocytes and (**c**) skeletal muscle tissue showing a reduction in AII-1 and AII-3 compared to control in canonical (blue), intron 5 retention (yellow) and all transcripts (green). CHX treatment was used to inhibit nonsense mediated decay in amniocytes. When normalising canonical and intron 5 retention transcript to all transcripts we observed a relative overrepresentation of intron 5 retention transcript in AII-3 amniocytes (**b**). Note that due to small amount of muscle tissue, we were unable to perform qPCR shown in (**c**) in triplicates and these results should be interpreted with caution. *p > 0.05, **p < 0.005, ***p < 0.001, ****p < 0.0001, one-way ANOVA with Šídák's multiple comparisons test.

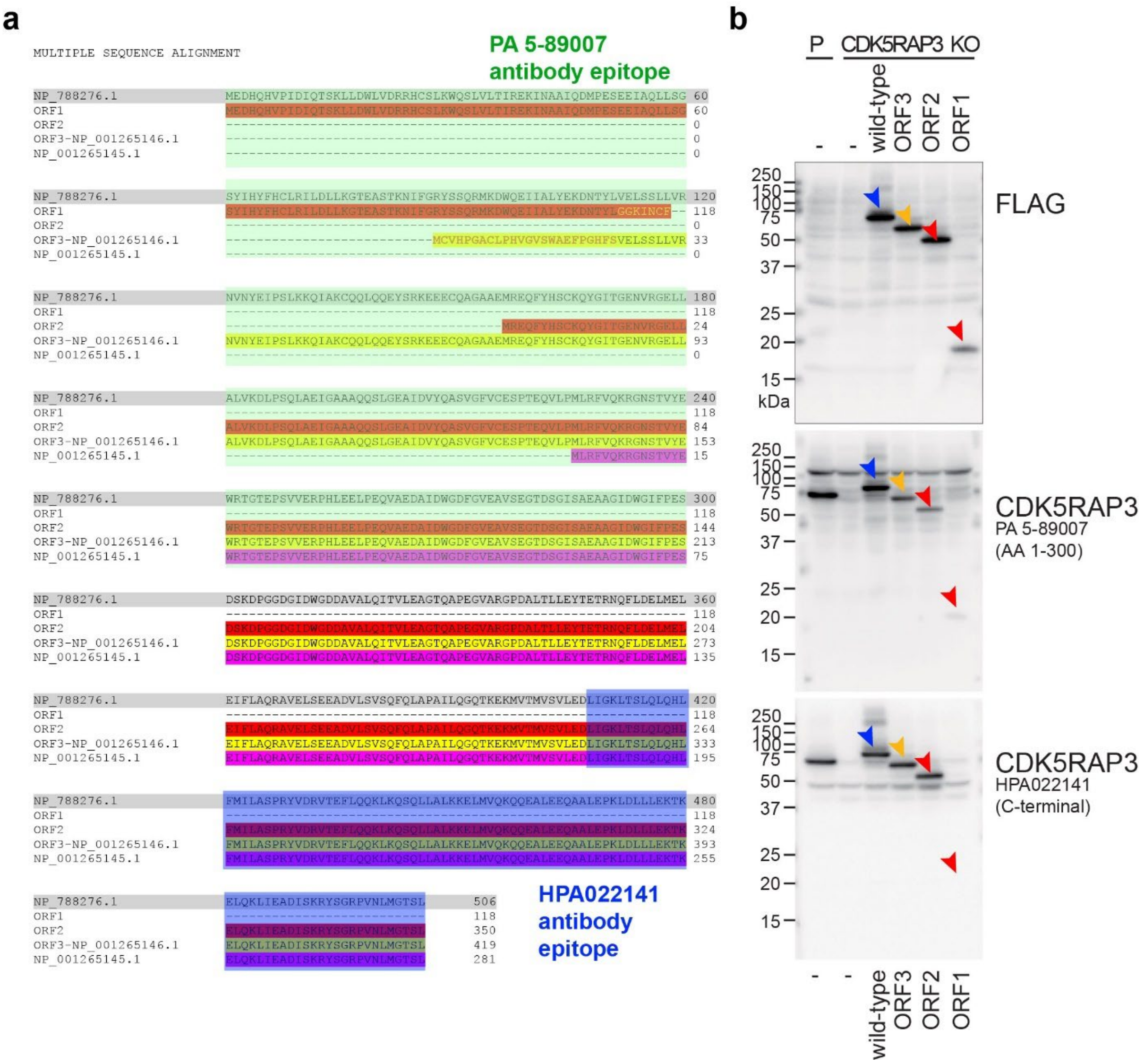

Fig. S3 Multiple sequence alignment of putative CDK5RAP3 isoforms and antibody validation

(**a**) Multiple sequence alignment created by CLUSTAL Omega (Version 1.2.4) of putative polypeptides translated from Open Reading Frames (ORF) present in *CDK5RAP3* transcripts. Canonical wild-type *CDK5RAP3* transcript NM_176096.3 encodes NP_788276.1 (56.93KD, grey highlight). NM_176096.3:c.334+243G>A induced PE inclusion between exon 5 and 6 is predicted to result in ORF1 and ORF2 (red highlight). A mis-spliced version of alternative *CDK5RAP3* transcript, NM_001278216.2, retaining part of intron 5 and all of intron 7, was also detected in AII-1 and AII-3. This transcript could give rise to an annotated short CDK5RAP3 protein isoform (31.21kD, NP_001265145.1, purple highlight) with a coding sequence unaffected by NM_176096.3:c.334+243G>A. A second alternative transcript, NM_001278217.2, which is identical to NM_001278216.2 except that it does not retain intron 7, encodes the putative protein NP_001265146.1 (46.36kD, yellow highlight) with a unique N-terminal sequence (orange text). (**b**) Western blot of *CDK5RAP3* knock-out cell lines transfected with FLAG-CDK5RAP3 wild-type (NP_788276.1, blue arrow), FLAG-ORF3 (NP_001265146.1, yellow arrow) and putative FLAG-ORF1 and FLAG-ORF2 resulting from PE inclusion (red arrow). Successful transfection was confirmed by probing with a FLAG-Antibody (top blot). Antibody PA5-89007 (raised against CDK5RAP3 amino acid 1-300, green

highlight) appears to detect ORF2 and ORF3 with reduced affinity compared to wild-type (middle blot). HPA022141 (raised against a C-terminal CDK5RAP3 peptide, blue highlight) detected wild type, ORF2 and 3 with similar affinity (bottom blot). PA5-89007 also detected the short N-terminal ORF1 while HPA022141 did not, confirming HP022141 binding a C-terminal CDK5RAP3 epitope.

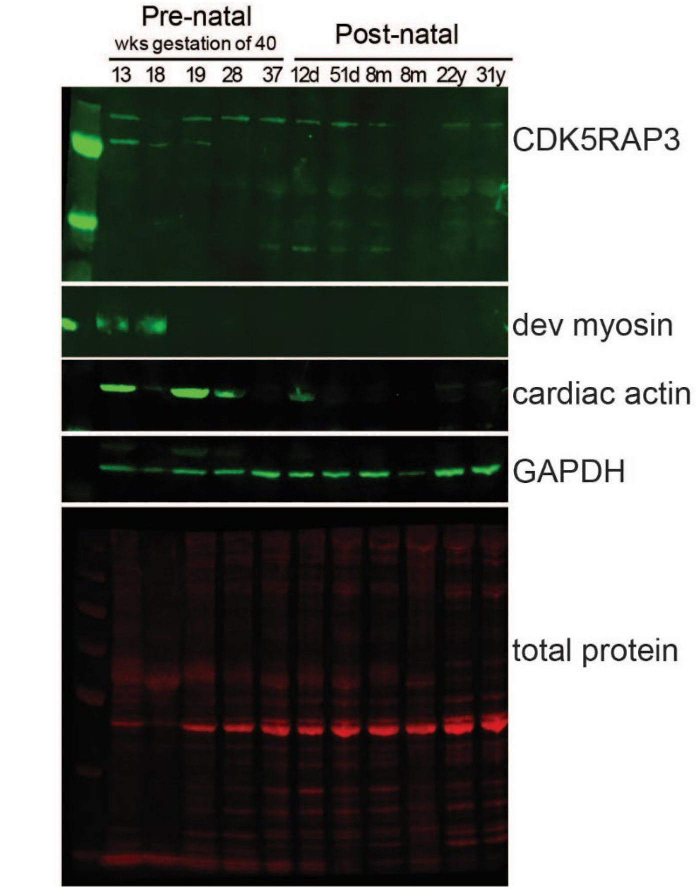

Fig. S4 Developmental expression of CDK5RAP3 in skeletal muscle

Western blot analysis of CDK5RAP3 (HPA022141) in skeletal muscle from individuals age 13/40 weeks gestation to 31 years. CDK5RAP3 is expressed throughout life expression appears higher in <8m compared to two adult controls. A second band is observed in samples <19/40 weeks gestation which may represent a protein product arising from alternative isoforms.

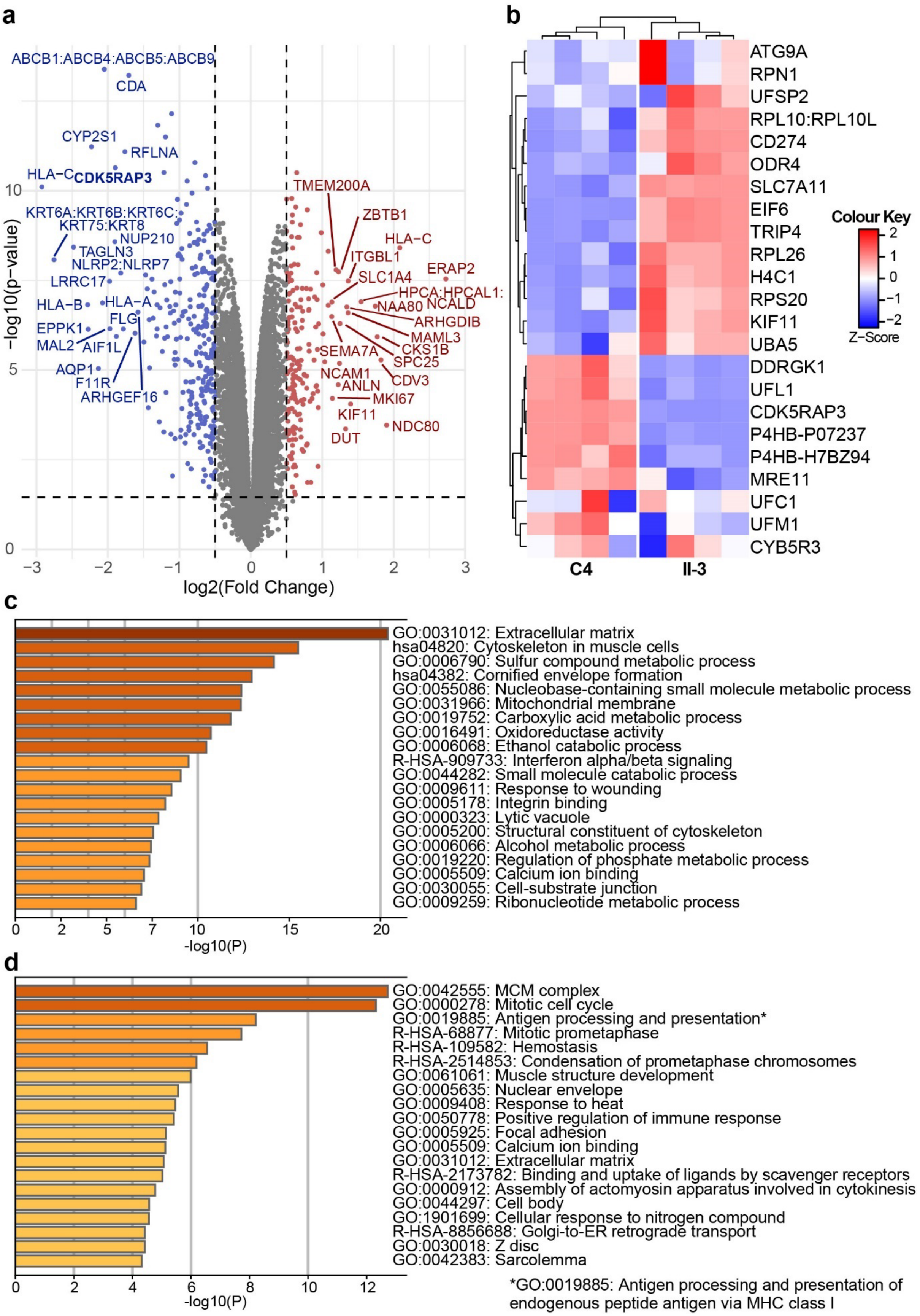

Fig. S5 Proteomics comparing affected sibling AII-3 and healthy primary control amniocytes (C4)

(**a**) Volcano plot with top 20 up- and downregulated proteins labelled. (**b**) Heat map showing the z-scores of UFMylation machinery and substrates. Metascape summary terms of top 20 (**c**) downregulated pathways and (**d**) upregulated pathways.

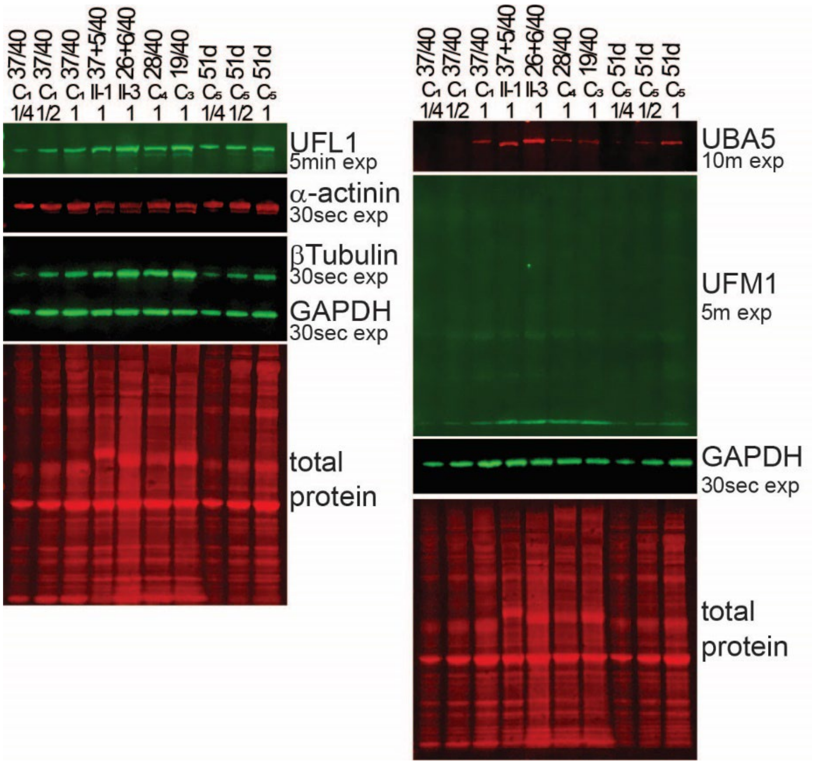

Fig. S6: UFL1, UBA5 and UFM1 expression in skeletal muscle

Western blot analysis of UFMylation pathway proteins UFL1, UBA5, UFM1 in skeletal muscle from affected siblings of Family A (AII-1, AII-3) and age matched controls. UFL1 expression was found to be similar to controls and UBA5 appears to be increased in affected siblings. UFM1 is not highly expressed in skeletal muscle but expression is comparable between affected siblings and controls. Bands of UFM1 conjugated proteins are not observed in skeletal muscle samples included here.

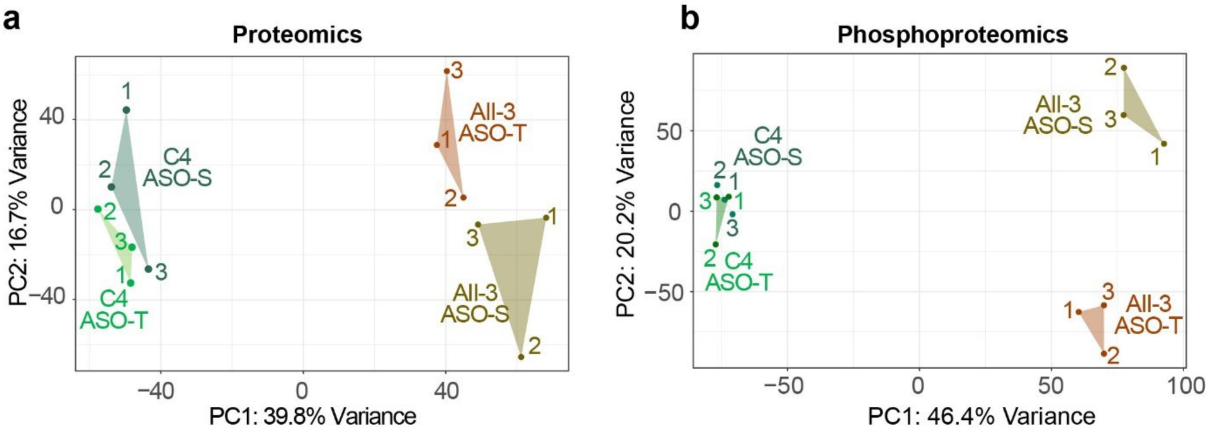

Fig. S7 Principal Components Analysis of proteomics and phosphoproteomics data

Principle Component Analysis following unwanted variant removal of (**a**) Proteomics and (**b**) Phosphoproteomics datasets showing clustering of control (C4) and AII-3 amniocytes treated with CDK5RAP3 targeting ASO (ASO-T) and scrambled ASO (ASO-S).

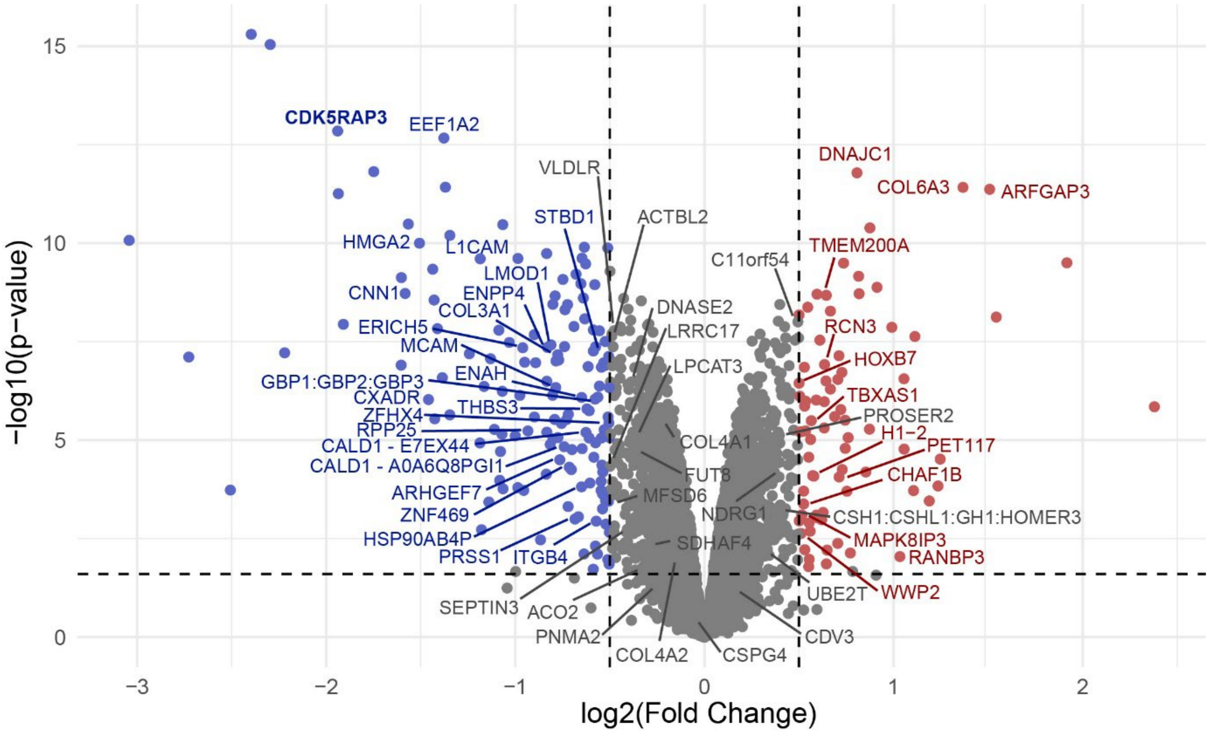

Fig. S8 Volcano plot of differential protein abundance between AII-3 and C4 scrambled ASO-treated amniocytes

With labels showing proteins up- and down-regulated by ASO-mediated CDK5RAP3 restoration (based on the comparison between AII-3 and C4 derived amniocytes treated with CDK5RAP3-targeting ASO-T; blue: significantly downregulated, red: significantly upregulated, grey: not significant)

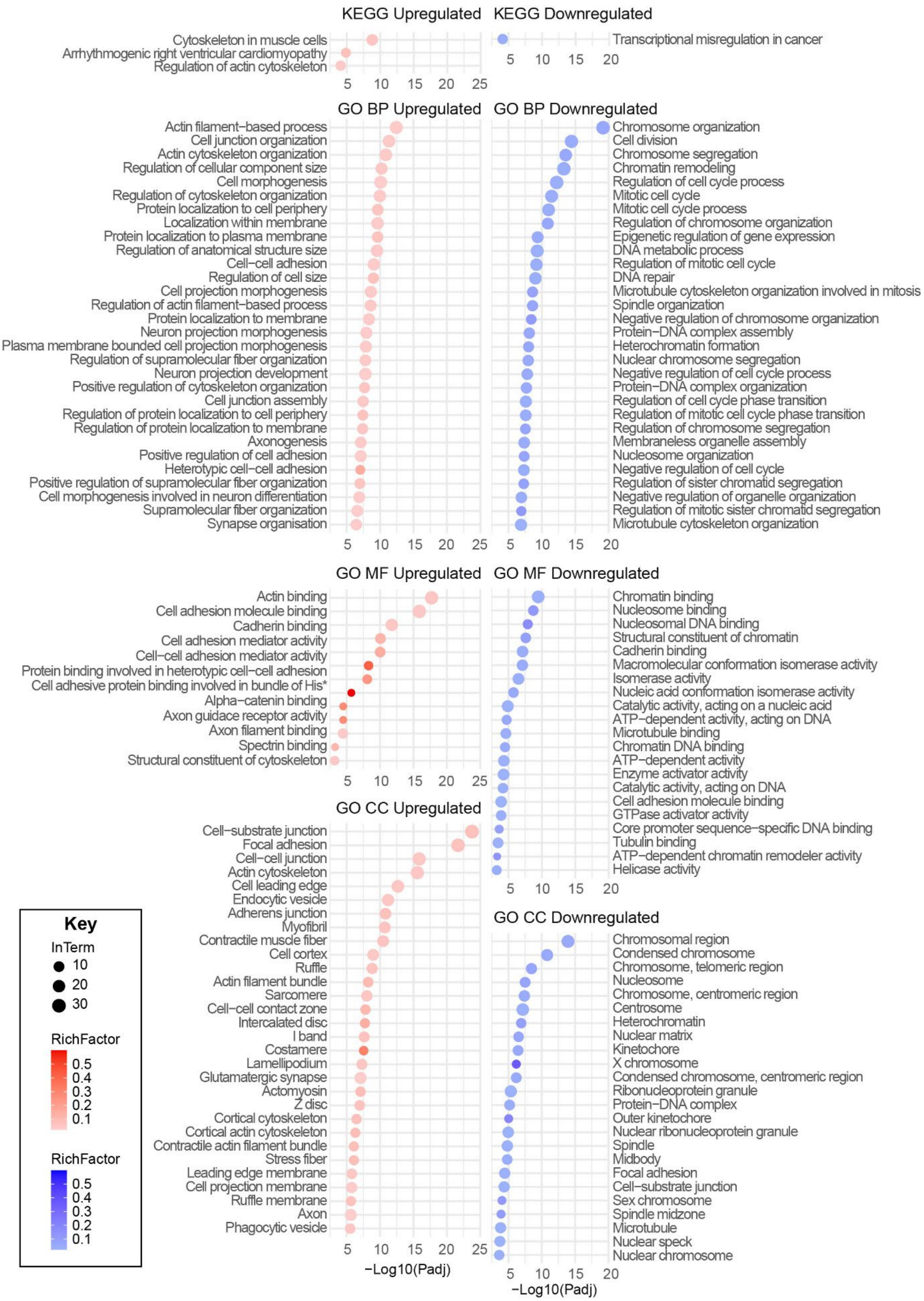

Fig. S9 KEGG/ GO terms from phosphoproteomics pathway analysis

Pathway enrichment analysis of the phosphoproteome 'ASO-T Rescue Set' revealed enrichment of 215 upregulated and 236 downregulated Reactome/KEGG/GO pathways

*Full GO term: Cell adhesive protein binding involved in bundle of His cell−Purkinje myocyte communication (truncated on figure for space consideration).

Table S1 Previously published cases with genetic disorders due to genetic variants in UFMylation pathway genes

UFSP2 cases

**Year**	**Publication**	**Mutation info**	**Disorder**	**Inheritance**	**Gene**
2015	**[38]**	NM_018359.5:c.868T>C (p.Tyr290His)	17 affected members of a family.<br>**Beukes hip dysplasia**.	Dominant<br>Heterozygous	*UFSP2*
2018	**[14]**	NM_018359.5:c.1277A>C (p.Asp426Ala)	3 affected members of a family.<br>**Beukes hip dysplasia**; progressive epiphyseal dysplasia; short stature, joint pain, genu vara and a novel spondyloepimetaphyseal dysplasia involving epiphyses predominantly at hips, but also at knees, ankles, wrists and hands, associated with variable degrees of metaphysis and spine involvement.	Dominant<br>Heterozygous	*UFSP2*
2020	**[41]**	NM_018359.5:c.1283A>G (p.His428Arg)	One affected individual.<br>**Spondyloepimetaphyseal dysplasia (SEMD)** manifested as: short stature, anterior vertebral dysplasia, hip dysplasia, flat vertebra, spinal metaphyseal dysplasia, irregular acetabular apex, distal femoral metaphyseal dysplasia, proximal tibial metaphyseal dysplasia, osteoarthritis.	Dominant<br>Heterozygous<br>*De novo*	*UFSP2*
2021	**[31]**	NM_018359.5:c.344T>A (p.Val115Glu)	8 affected children from 4 families.<br>**Neurodevelopmental delay and epilepsy.**	Recessive	*UFSP2*

2023	**[34]**	NM_018359.5:c.344T>A (p.Val115Glu)	2 affected siblings.<br><br>**West syndrome/infantile spasm syndrome,** a type of **early epileptic encephalopathy**. Characterised by early onset drug-resistant seizures with persistent EEG abnormalities and cognitive deficits.	Recessive	*UFSP2*
2023	**[25]**	NM_018359.3:c.905G>C (p.Cys302Ser)	1 affected individual.<br><br>**Spondyloepimetaphyseal dysplasia (SEMD)** presenting with disproportionate short stature, genu varum, gait instability, epiphyseal and metaphyseal alterations, bell-shaped thorax, lumbar hyperlordosis, muscular hypotonia, and coxa vara.	Dominant<br><br>Heterozygous<br><br>*De novo*	*UFSP2*

UBA5 cases

**Year**	**Publication**	**Mutation info**	**Disorder**	**Inheritance**	**Gene**
2016	**[29]**	NM_024818.6:c.1111G>A (p.Ala371Thr) (allele frequency of 0.28% in Europeans)<br><br>With a nonsense variant<br><br>or<br><br>NM_024818.6:c.164G>A (p.Arg55His) (splice altering - exon 2 skipping)	9 affected individuals from 5 families.<br><br>**Early infantile-onset encephalopathy**. Presenting in infancy with irritability followed by dystonia and stagnation of development. Epileptic seizures, intellectual deficits, progressive microcephaly, growth failure, truncal hypotonia, spasticity.	Recessive<br><br>Compound heterozygous	*UBA5*

2016	**[11]**	Family A: NM_024818.6:c.904C>T (p.Gln302Ter), NM_024818.6:c.1111G>A (p.Ala371Thr)<br><br>Family B: NM_024818.6:c.1111G>A (p.Ala371Thr), NM_024818.6:c.971_972insC (p.Lys324fs)<br><br>Family C: NM_024818.6:c.778G>A (p.Val260Met), NM_024818.6:c.1165G>T (p.Asp389Tyr)<br><br>Family D: NM_024818.6:c.169A>G (p.Met57Val), NM_024818.6:c.503G>A (p.Gly168Glu)	5 affected children from 4 unrelated families.<br><br>**Early-onset severe neurological disorder** featuring severe intellectual deficiency, microcephaly (5/5), movement disorder (3/5), early onset intractable epilepsy (3/5). Brain MRI showing thin corpus callosum (3/5), cerebellar atrophy (2/5)	Recessive<br><br>Compound heterozygous	*UBA5*
2016	**[15]**	NM_024818.6:c.736C>T (p.Arg246Ter), NM_024818.6:c.928A> G (p.Lys310Glu)	2 affected siblings.<br><br>**Progressive cerebellar ataxia** featuring gait and limb ataxia, speech difficulties, cerebellar atrophy, cataracts; marked cerebellar atrophy and loss of ambulance in one affected individual, mild ataxia and an associated demyelinating peripheral neuropathy in the other. Both patients reached adulthood. Neither had cognitive impairment.	Recessive<br><br>Compound heterozygous	*UBA5*

2017	**[3]**	Paternal: NM_024818.6c.684G>A (p.Ala228=) predicted to disrupt exon 7 splice site, resulting in loss-of-function. Maternal: NM_024818.6:c.1111G>A (p.Ala371Thr)	2 affected siblings.<br>**Early-onset epileptic encephalopathy:** infantile spasms from 6 months of age, progressing to recurrent, treatment-resistant seizures. First symptoms at 3 months of age (failure to thrive, decline in motor skills such as tracking and head support). Severe intellectual disability with seizures and dystonic cerebral palsy.	Recessive<br>Compound heterozygo us	*UBA5*
2018	**[26]**	NM_024818.6:c.158A>T (p.Tyr53Phe)	One affected individual.<br>**Early myoclonic epilepsy (EME)/Aicardi syndrome:** severe global hypotonia, epileptic manifestation from 3 hours after birth (clonic movements of the upper limbs). No MRI or metabolic abnormalities. The affected individual died at day 16 after cardiac arrest due to respiratory failure from status epilepticus.	Recessive<br>Homozygo us	*UBA5*
2018	**[13]**	NM_024818.6:c.214C>T (p.Arg72Cys)<br>Microdeletion of ~3.2 Mb within the 3q22.1 region encompassing UBA5 (Chr3:129762317-132948291)	One affected individual.<br>**Intractable West syndrome**, profound failure to thrive, and severe cerebral and cerebellar atrophy. The affected individual presented with epileptic spasms and hypsarrhythmia at 3 months. MRI findings were initially normal but subsequently showed a progression of cerebellar and cerebral atrophy. No achievement of any developmental milestones by 7 years, daily epileptic spasms and tonic seizures and profound failure to thrive.	Recessive<br>Compound heterozygo us	*UBA5*
2018	**[10]**	NM_024818.6:c.1214A>T (p.Ter405Leu), NM_024818.6:c.1111G>A (p.Ala371Thr)	3 affected individuals of a family.<br>**Progressive encephalopathy, hypsarrhythmia and optic atrophy (PEHO)-like syndrome**: truncal	Recessive	*UBA5*

			hypotonia, spasticity, infantile spasms and epileptic seizures, secondary microcephaly, brain MRI showed mild cerebral or cerebellar atrophy, all but one had severe developmental delay.	Compound heterozygous	
2019	**[24]**	De novo 597 kb deletion on 3q22.1 described as: arr[GRCh37]3q22.1 (131,875,931_132,472,766) ×1 dn which includes the whole UBA5 gene,<br><br>NM_024818.6:c.1166A>G (p.Asp389Gly)	One affected individual.<br><br>**Early onset epileptic encephalopathy** featuring significant developmental delays in all areas, infantile spasms and hypsarrhythmia starting at 4 months, epileptic spasms, tonic seizures and possible myoclonic seizures, acquired microcephaly, dyskinetic movement disorder. Head control not achieved at age 3.5 years. Brain MRI showed non-progressive lack of volume within the cerebellum with delayed myelination pattern.	Recessive<br><br>Compound heterozygous	*UBA5*
2020	**[7]**	NM_024818.6:c.31C>T (p.Arg11Trp)	**Severe congenital neuropathy** causing early death in infancy (respiratory failure 19 days to 16 weeks). Irritability, severe weakness including facial weakness, hypotonia, poor feeding, motor delay. Reduced foetal movement. Foot and ankle deformities and contractures from birth. Some antigravity movements initially but progressive weakness. Brain/spine MRI and EEG were normal. Nerve conduction studies were abnormal.	Recessive<br><br>Homozygous	*UBA5*
2020	**[27]**	NM_024818.6:c.160dupA (p.Ser54LysfsTer16),<br><br>NM_024818.6:c.215G>A (p.Arg72His)	One affected individual.<br><br>**Epileptic encephalopathy, early infantile**	Recessive<br><br>Compound heterozygous	*UBA5*

2021	**[6]**	All have the recurrent mild NM_024818.6:c.1111G>A (p.Ala371Thr) variant in trans with a second *UBA5* variant:<br><br>- 1 has the previously described NM_024818.6:c.562C>T (p.Arg188Ter)<br><br>- 2 unrelated have a novel variant NM_024818.6:c.907T>C (p.Cys303Arg)<br><br>- two siblings have a novel variant NM_024818.6:c.761T>C (p.Leu254Pro)	5 affected individuals from 4 families.<br><br>**Early infantile epileptic encephalopathy** featuring global developmental delay, epilepsy, axial hypotonia, appendicular hypertonia.<br><br>P1: global developmental delay, cortical visual impairment, seizures, axial hypotonia, appendicular hypertonia and dystonia, and failure to thrive. Seizures from 6 years (drug resistant). Brain MRI normal at 5 months but posterior thinning of corpus callosum at 22 months, patchy T2 signal prolongation in the white matter, and incompletely rotated hippocampi.<br><br>P2: severe global developmental delay, failure to thrive, mild microcephaly, axial hypotonia, appendicular hypertonia and spasticity, and a movement disorder consisting of severe dystonia and mild chorea. Seizures from 5 years.<br><br>P3: global developmental delay, intellectual disability, epilepsy, axial hypotonia, appendicular hypertonia, ataxia, strabismus, failure to thrive, and microcephaly. Seizures from 4 years. Brain MRI normal at 3 and 8 years, microcephaly and decreased frontal white matter parenchymal volume at 15 years.<br><br>P4: global developmental delay, intellectual disability, drug-resistant multifocal and generalized epilepsy, hypotonia, mild spasticity, dystonia, failure to thrive, microcephaly, astigmatism, and myopia. Infantile spasms	Recessive<br><br>Compound heterozygous	*UBA5*

			were first noted at 7 months. Non-ambulatory and nonverbal. Brain MRI at 12 years was normal.<br>P5: global developmental delay, intellectual disability, seizures, hypotonia, spasticity, dystonia, nonepileptic myoclonus, mild microcephaly, and cortical visual impairment. Two brain MRIs, the most recent at 6 years, were reportedly normal.		
2021	**[1]**	NM_024818.6:c.895C>T (p.Pro299Ser)	One affected individual.<br>**Severe global developmental delay and epilepsy**	Recessive<br>Homozygous	*UBA5*
2023	**[39]**	NM_024818.6:c.1111G>A(p.Ala371Thr)<br>NM_024818.6:c.110C>T (p.Thr37Ile)	One affected individual.<br>**Developmental epileptic encephalopathy** with intellectual disability (non-verbal), status dystonicus, axial hypotonia, spastic tetraparesis, mild generalized dystonia, dysphagia.	Recessive<br>Compound heterozygous	*UBA5*
2025	**[9]**	NM_024818.6:c.1111G>A (p.Ala371Thr) with<br>Individual 1:<br>NM_024818.6:c.367_368insGA (p.Ala123fs)	2 affected individuals from different families.<br>**Developmental epileptic encephalopathy** featuring intractable epilepsy, severe developmental delay, spasticity, cortical visual impairment<br>P1: Early delays with feeding difficulties shortly after birth, poor head control, hypotonia, and decreased	Recessive<br>Compound heterozygous	*UBA5*

		Individual 2:<br>NM_024818.6:c.799C>T (p.Gln267Ter)	engagement in the first few months after birth. Developed refractory seizures at 3 months. MRI showed a thin corpus callosum and delayed myelination. He makes sounds but is nonverbal; he uses eye gaze for "yes" and "no" communication. He is nonambulatory but uses a stander with assistance<br>P2: Developed infantile spasms at 4 months. Later developed refractory tonic seizures, myoclonic jerks, and atypical absence seizures. MRI showed thinning of the corpus callosum and slowly progressive cerebral volume loss. Microcephaly, with head circumference below the third percentile since at least 6 months old. She has minimal interaction; is nonverbal; and is unable to sit, stand, or walk independently.		

UFM1 cases

Year	Publication	Mutation info	Disorder	Inheritance	Gene
2017	**[20]**	NM_016617.4:c.-155_-153delTCA<br>Deletion in the promoter region	16 affected individuals with founder mutation.<br>**Leukodystrophy featuring hypomyelination with atrophy of the basal ganglia and cerebellum**. Severe developmental delay, typically without intentional movements and language development, microcephaly, spasticity and extrapyramidal movement abnormalities, severe and drug-resistant epilepsy. Median age of onset is 2 months. 94% had no intentional movements and no language. Death between 7 months and 7 years (respiratory insufficiency)	Recessive<br>Homozygous	*UFM1*

2018	**[30]**	NM_016617.4:c.241C>T (p.Arg81Cys)	4 affected individuals from 2 families.<br>**Severe early-onset encephalopathy with progressive microcephaly,** profound global developmental delay, failure to thrive, short stature, progressive microcephaly and refractive epilepsy. MRI showed delayed CNS myelination (3/4) and cerebellar hypoplasia (3/4).	Recessive<br>Homozygous	*UFM1*
2020	**[8]**	NM_016617.4:c.-155_-153delTCA	One affected individual.<br>**Hypomyelinating leukodystrophy**	Recessive<br>Homozygous	*UFM1*
2021	**[36]**	NM_016617.4:c.-155_-153delTCA	4 affected individuals.<br>**Hypomyelinating leukodystrophy** featuring severe global developmental delay, axial hypotonia, spasticity, regression. 1/4 had seizures but all had diffuse cortical dysfunction on MRI, brain atrophy (cortex and basal ganglia), delayed/absent myelination, cerebellar hypoplasia. Median survival was 28 months.	Recessive<br>Homozygous	*UFM1*
2021	**[5]**	NM_016617.4:c.-155_-153delTCA	One affected individual.<br>**Progressive neurodegenerative disease** at age 7 years, presenting with profound intellectual disability and epilepsy, history of apneic episodes and feeding difficulties in infancy.	Recessive<br>Homozygous	*UFM1*
2023	**[23]**	NM_016617.4:c.-155_-153delTCA	9 affected individuals.	Recessive	*UFM1*

			**Hypomyelination with atrophy of the basal ganglia and cerebellum.** Presentation at < 2 months (inspiratory stridor, impaired sucking, swallowing, vision and hearing, and reduced active movements), microcephaly (6/9), malnutrition (5/9), muscle hypertonia (9/9) and axial hypotonia (4/9), progressing to opisthotonus (6/9), dystonic posturing (5/9), nystagmoid ocular movements (6/9), epileptic seizures (4/9), non-epileptic spells (3/9). Dysphagia (7/9), inspiratory stridor (9/9), dyspnea (5/9), bradypnea (5/9), apnea (2/9). Vision and hearing were never achieved or lost by 4-8 months. Neurodevelopment was absent or minimal with subsequent regression after 2-5 months. Brain imaging revealed cortical atrophy (7/9), atrophic ventricular dilatation (4/9), macrocisterna magna (5/9), reduced myelination (6/6), corpus callosum atrophy (3/6) and abnormal putamen and caput nuclei caudati. The age at death was between 8 and 18 mo.	Homozygous	

DDRGK1 cases

**Year**	**Publication**	**Mutation info**	**Disorder**	**Inheritance**	**Gene**
2017	**[16]**	NM_023935.3:c.408+1G>A<br><br>donor splice site loss-of-function mutation	7 affected individuals from 4 families.<br><br>**Shohat-type spondyloepimetaphyseal dysplasia**	Recessive<br><br>Homozygous	*DDRGK1*

2022	**[17]**	NM_023935.3:c.406G>A (p.Glu136Lys)	2 affected individuals from 2 families.<br><br>**Shohat-type spondyloepimetaphyseal dysplasia**	Recessive<br><br>Homozygous	*DDRGK1*
2022	**[32]**	NM_023935.3:c.408+1G>A	One affected individual.<br><br>**Shohat-type spondyloepimetaphyseal dysplasia**. Additionally, left choanal atresia and prenatal fractures (not previously described with SEMD Shohat-type), microcephaly, and beaten sliver skull on X-ray not present at birth. Normal cognitive function.	Recessive<br><br>Homozygous	*DDRGK1*

***UFC1* cases**

**Year**	**Publication**	**Mutation info**	**Disorder**	**Inheritance**	**Gene**
2017	**[2]**	NM_016406.3:c.317C>T: p.(Thr106Ile) homo	2 affected individuals from a family.<br><br>**Intellectual disability.**<br><br>P1: Floppiness since birth with delay in milestones. spastic at 4 years, seizures from 13 years. No head control, wheelchair bound, speech is limited to babbling. Neurological assessment at 13 years showed spasticity, hyperreflexia, mild intention tremor and hirsutism. Brain MRI showed white matter changes.<br><br>P2: global developmental delay, spasticity and ataxia. Neurological examination at 10 years showed hypertonia, and hyperreflexia with clonus. Wheelchair	Recessive<br><br>Homozygous	*UFC1*

			bound, intention tremor and oculomotor apraxia. Brain MRI at 14 years was unremarkable.		
2018	**[30]**	3 families with NM_016406.3:c.317C>T (p.Thr106Ile)<br><br>1 family with NM_016406.3:c.68G>A (p.Arg23Gln)	8 affected individuals from 4 families (1 already described in Anazi et al 2016)<br><br>**Severe early infantile encephalopathy**, progressive microcephaly (7/8), axial hypotonia, appendicular hypertonia and refractory epilepsy (4/8). MRI showed basal ganglia abnormality (2/6) and delayed CNS myelination (1/6).	Recessive	*UFC1*
2019	**[33]**	NM_016406.3:c.68G>A (p.Arg23Gln)	One affected individual.<br><br>**Early-onset epileptic encephalopathy** and combined **developmental epileptic encephalopathy**	Recessive<br><br>Homozygous	*UFC1*
2024	**[21]**	NM_016406.4:c.19C>T (p.Arg7Ter), NM_016406.4:c.164G>A (p.Arg55Gln)	One affected individual.<br><br>**Global neurodevelopmental delay** (severely delayed psychomotor development not standing or talking at 17 months), microcephaly, epilepsy, severely delayed psychomotor development, severe growth retardation facial deformities. MRI showed diffuse white matter loss suggestive of cerebral dysplasia. Infantile spasms from 3 months.	Recessive<br><br>Compound heterozygous	*UFC1*

2025	**[40]**	NM_016406.4: c.141del, p.Asn48Metfs*29/c.255+17G>A;het/het, indel/SNV, 1bp/1bp<br>NM_016406.4: c.244_255del, p.Glu83_Ile86del/c.255+17G>A;het/het, indel/SNV, 12bp/1bpv	Neurodevelopmental disorder with spasticity and poor growth (infantile-onset dystonia, segmental; spasticity, DD (spastic-dystonic CP))<br>Neurodevelopmental disorder with spasticity and poor growth, (infantile-onset dystonia, generalised; spasticity, DD, epilepsy, microcephaly)	Recessive<br>Recessive	*UFC1*

Table S2 Primer, PCR and ASO information

**Primer name**	**Sequence 5'-3'**	**Annealing temp (°C)**	**Amplicon (bp)**	**PCR conditions**
Sanger segregation primers<br>*CDK5RAP3*_Int5_gFW<br>*CDK5RAP3*_Int5_gRV	GATGTTGCCCATTTGTAGGTG<br>TACACCCAACCCAAACACAG	60	297	MyTaq polymerase (Meridian Biosciences, Cincinnati, Ohio, USA) as per manufacturer's protocol. PCR cycling conditions: 95 °C 2 min; 35 cycles of 95 °C 10 s, 60 °C 30 s, 72 °C 1 min; final 72 °C 8 min
*GAPDH*_Ex3_FW<br>*GAPDH*_Ex6_RW	TCACCAGGGCTGCTTTTAAC<br>GGCAGAGATGATGACCCTTT	64	317	Extension time: 90s
*CDK5RAP3*_Ex4-F<br>*CDK5RAP3*_Ex11-R	CCTTCTCAAAGGCACAGAGG<br>AGTGTATTCAAGCAGTGTCAGG	58	NM_176096.3: 823bp<br>NM_001278217.2: 1533<br>NM_001278216.2: 1748	Extension time: 240 s (Long Amp Taq, NEB)
*CDK5RAP3*_Ex4-F<br>*CDK5RAP3*_Ex5/6-R	CCTTCTCAAAGGCACAGAGG<br>GGAGGCTAGAGAGTTCCACTAAG	60	134	Extension time: 60s (qPCR) PowerUp SYBR Green master mix; manufacturer-specified cycling conditions

*CDK5RAP3*_Ex4-F<br>*CDK5RAP3*_Ex5-R	CCTTCTCAAAGGCACAGAGG<br>TGTTGTCCTTCTCATACAGAGC	60	121	Extension time: 60s (qPCR) PowerUp SYBR Green master mix; manufacturer-specified cycling conditions
CDK5RAP3_Ex4-F<br>CDK5RAP3_I5-R	CCTTCTCAAAGGCACAGAGG<br>GCCTCCTATAAGCCTGGTATC	60	198	Extension time: 60s (qPCR) PowerUp SYBR Green master mix; manufacturer-specified cycling conditions
HPRT1_F<br>*HPRT1_R*	TCCAAAGATGGTCAAGGTCGC<br>TTCAAATCCAACAAAGTCTGGCT	60	85	Extension time: 60s (qPCR) PowerUp SYBR Green master mix; manufacturer-specified cycling conditions
RPLP0_F<br>*RPLP0_R*	CATGCTCAACATCTCCCCCT<br>ACCCTCCAGGAAGCGAGAAT	60	127	Extension time: 60s (qPCR) PowerUp SYBR Green master mix; manufacturer-specified cycling conditions
GAPDH_F<br>*GAPDH_R*	GGTCGGAGTCAACGGATTTGG<br>TTCTCAGCCTTGACGGTGC	60	177	Extension time: 60s (qPCR) PowerUp SYBR Green master mix; manufacturer-specified cycling conditions PowerUp SYBR Green master mix; manufacturer-specified cycling conditions
XBP1_common_F<br>*XBP1_common_R*	CCTGGTTGCTGAAGAGGAGG<br>ATCCATGGGGAGATGTTCTGG	58	121/147	Extension time: 30 s
XBP1s_F<br>*XBP1s_R*	TGCTGAGTCCGCAGCAGGTG<br>GCTGGCAGGCTCTGGGGAAG	58	169	Extension time: 30 s
XBP1u_F<br>*XBP1_common_R*	CAGCACTCAGACTACGTGCA<br>ATCCATGGGGAGATGTTCTGG	58	76	Extension time: 30 s
CDK5RAP3 ASO (ASO-T)	ACCCACACATACCTGCTCGCTGAG	-	-	-
SCR ASO (ASO-S)	GCTCAACTGCCGCAACCCTTACGA	-	-	-

Table S3 Full autopsy results for affected individuals from Family A

**Organ**	**AII-1** (*expected weight at 37+5/40 weeks gestation in bracket)	**AII-3** (*expected weight at 26+6/40 weeks gestation in bracket)
Brain	190 g (343.6 ± 37.9 g)	91.9 g (124.9 ± 18.2 g)<br>Brain/Liver ratio 3.6 (>5 in intrauterine growth restriction)
Dural Meninges and leptomeninges	Oedematous and cloudy	Normal
Cerebrum	• Structurally normal, symmetrical with atrophic gyri and prominent sulci and a normal cortical ribbon.<br>• No neural migration defects observed (e.g. no periventricular neuronal heterotopia, polymicrogyria or simplification of the cerebral architecture).<br>• Normal lamination present.<br>• Widespread blurring of the grey-white matter junction with preserved grey/white matter proportion.<br>• The cerebral cortical mantle was preserved, and the cortex had a normal layered pattern with increased glial cells and reactive astrocytes.<br>• Subcortical white matter is variably oedematous and there are reactive astrocytes including gemistocytic forms (GFAP positive) as well as increased numbers of CD68 microglia mainly in deeper white matter.	• Two symmetrical cerebral hemispheres.<br>• The basal ganglia are identified and appear normally formed and symmetric.<br>• The germinal matrix is thick and preserved.<br>• The hippocampus is normal.
Midbrain	• The corpus callosum was intact and normal in (some reactive astrocytes).	• The corpus callosum was intact and normal.<br>• Thalamus size was not noted.

	• The thalamus, basal ganglia, hippocampus, ventricular lining, and choroid plexus were normally developed (except for the 4x3mm cyst, **Figure 1 B, C**).<br>• Thalamus size was reduced.	
Cerebellum, pons and medulla	• Smaller even compared to reduced brain size.<br>• Overall normal architecture.<br>• The external granular and molecular layer have normal appearance, but Purkinje cell layer has normal number of Purkinje cells in vermis but reduced or lacking Purkinje cells in the cerebellar hemispheres.<br>• Internal granular cell layer is present but appears to contain fewer cells than typical in much of the cerebellum.<br>• On H&E some areas of cerebellar hemispheres show areas suspicious for Bergmann's gliosis and small amounts of GFAP positive staining is seen in these regions (not pronounced).<br>• The dentate nucleus is abnormally simplified with nodular pattern and reduced number of neurons (similar to olives) and increased number of glial cells in the tissue surrounding the dentate.<br>• Poorly defined olives, fewer than normal staining neurons and increased numbers of glial cells on GFAP.	• The brainstem and cerebellum are abnormal, but the grey matter nuclei and structures are symmetric.<br>• The cerebellum is small, and the foliation of the hemispheres is simple, with relative preservation of the foliae of the vermis.<br>• The cortical lamination of the cerebellum is normal, with a single nodular focus of GFAP positive cells in the cerebellar cortex representing an area of gliosis but overall preservation of the cortex.<br>• Probably delayed Purkinje cell migration (Purkinje cells only seen in the most inferior part of the vermis, and none in the hemispheres).<br>• Dentate nucleus identified, but slightly less convoluted than expected, with isolated nodules seen towards the midline, but no interruption of the contour of the dentate.<br>• The pontine nuclei appear smaller than expected for gestational age. The pontine transverse fibres are present, but there are less neurons than expected.<br>• The pyramids appear normally sized but abnormally shaped.<br>• The inferior olivary nucleus is abnormally formed (minimal convolution, simple "U" shape, multiple discontinuous nodules at the peripheral aspect).<br>• The remainder of the brainstem nuclei were smaller than expected but appeared symmetric and normally formed.
Cranial nerves	Normal	Normal
Eyes	Normal	Normal

Facial features	• Nose is normally sited and formed with patent posterior nasal choanae.<br>• The philtrum and tongue are normal.<br>• Narrow mouth.<br>• Intact palate but hard palate has a high arched appearance and soft palate is normal<br>• Chin appears small.<br>• Ears are normally sited with well-formed pinnae and patent external auditory canals.	• Nose is normally sited but has prominent alae nasi and columella with notching of the nostril. The posterior nasal choanae are patent, and there is a high arched palate with no cleft.<br>• The philtrum and tongue are normal.<br>• Mouth is downturned<br>• Chin is micrognathic.<br>• Eyes are protuberant with deep infraorbital creases and flattened brow ridges.<br>• Ears are normally sited with well-formed pinnae and patent external auditory canals. No pre/post-auricular pit or sinuses.
Digestive tract	• Oesophagus normal.<br>• No comment on remaining digestive tract.	• Oesophagus normal.<br>• Meconium present in small and large intestine.<br>• Stomach, small intestine and colon are patent through to the anus with no evidence of malrotation or atresia.<br>• The caecum and appendix are located in the right lower quadrant.
Liver	59.5 g (108.4 ± 1.2 g)<br>• Ductular reaction, extramedullary haematopoiesis (probably more than typical for corrected gestational age) and probably a mixed chronic inflammatory infiltrate of mainly lymphocytes and a few plasma cells.<br>• The portal areas are irregularly expanded by the fibrosis, giving stellate appearance and in many areas, there is portal-to-portal bridging, but portal to central bridging is not seen. A few areas show almost complete nodule formation.<br>• Interlobular bile ducts, hepatic arterioles and portal venules are present within the portal tracts.	25.4g (45.9 ± 1.2 g)<br>• Liver appears fragile and haemorrhagic.<br>• There is a normal gallbladder and extrahepatic bile ducts.<br>• There is moderate autolysis, but the lobular-acinar architecture is preserved.<br>• The biliary duct development is difficult to assess due to autolysis, but on cytokeratin stain there are ducts seen in the larger portal tracts, and focal persistence of the ductal plate.<br>• Early cholestatic changes were present in the liver including bile plugs, which is likely to be significant at this gestational age, but is of unclear aetiology given the normal ductal plate and biliary development.

	• No significant bile duct damage but occasional bile duct plugging. The limiting plate is mildly irregular, partially obscured mainly by the extramedullary haematopoiesis and ductular reaction rather than hepatocyte dropout.<br>• Hepatocytes show a normal sinusoidal trabecular pattern and there is mild intrahepatocytic and bile canalicular cholestasis.<br>• Keratin stain (MCK) shows a moderate intensity periportal bile ductular proliferative reaction.	• Extramedullary haematopoiesis is seen and there is no calcification, but the morphology of the hepatocytes is partly lost due to autolysis.
Pancreas	1.6 g (2.0 ± 1.7 g)	0.7 g (0.7g ± 1.8 g)
Thymus	14.9 g (7.5 ± 1.6 g)<br>Prominent appearance, pale colour, normally located, normal ultrastructure; 4-5mm nodule identified as thymic tissue between the superior aspect of the thymus and the inferior pole of the thyroid.	2.3 g (2.0 ± 1.6 g)<br>Normally located and formed thyroid gland.
Spleen	13 g (7.7 ± 1.4 g)<br>Normal developed red and white pulp for the gestational age. the spleen is unremarkable, except for congested red pulp.	1.2 g (1.6±1.4g)<br>Red and white pulp differentiation is preserved. Lymphoid aggregates are identified. No viral cytopathic changes are seen.
Kidneys	R: 8.3g (12.3±2.8g) and L: 7.8g (12.4 ±2.6g)<br>Normally developed. Showed some amorphous basophilic mineral deposits within tubular lumens, suggesting one or more previous episodes of renal tubular damage, but no evidence of active/current damage. Incidental finding of dual right renal arteries.	R: 2.3g (4.4±1.1g) and L: 2.2g (4.5±1.1g)<br>Autolysed. Preservation of corticomedullary differentiation and a normal cortical nephrogenic zone. No cystic change or dilatation of the pelvis. No obvious dysplastic features including cartilage or stromal nodules. There is loss of tubular nuclear basophilia.
Adrenals	2.9 g (6.9±2.1g)	1.2g (2.7± 0.9g)
Lungs and airways	Lung/body weight ratio of 1.32 % (; < 1.15 % in hypoplasia)	Lung/body weight ratio of 1.96 % (< 1.15 % in hypoplasia)<br>R: 6.7g (11.8±2.8g) and L: 5.1g (9.7±2.2g)

	R: 15.6 g (28.9±5.8g) and L: 10.5 g (23.7±5.8g)<br><br>Larynx, trachea, bronchi and lung developed normally.	No definite hypoplasia<br>Larynx, trachea, bronchi and lung developed normally. The small lungs are likely to be secondary to decreased intrauterine diaphragmatic and intercostal movement rather than a primary hypoplastic process.
Heart	8.5 g (17± 3.8g)<br>Patent foramen ovale and 1 mm atrial septal defect	3.9 g (5.7±1.5g)<br>Normal morphology
Placenta	N/A	Placental disc weight: 124.5 g (226±100g), Umbilical cord length 375mm (425±113mm)<br>• Underweight placenta; multiple foci of villous calcification (of uncertain significance, likely an incidental finding).<br>• Area of haemorrhagic infarction (recent and most likely secondary to induction of labour).<br>• A single focus of basal villitis is seen (of uncertain significance, likely of minimal contribution).
Genitals and reproductive organs	Left testis, poorly formed scrotum	Normal
Skeletal muscle	• Muscles appeared macroscopically normal, including the diaphragm, intercostal muscles, and strap muscles of the neck.<br>• The psoas muscle was smaller but proportional to the smaller baby, while the biceps were normal.<br>• The quadriceps appeared more fatty and ill-defined, possibly fibrotic.<br>• Clusters of small myofibres in some areas.<br>• Muscle fibers that are larger (~22µm) or smaller (~4.5µm) than expected for gestational age.<br>• Slightly increased number of cells with internal nuclei (3%).	• Muscle wasting which is more severe in the distal limbs.<br>• No overt features of either myopathic or neuropathic change.<br>• There was no evidence of primary hypoplasia or atrophy. The average fibre size is 17.8 µm (range from 7.5-30.9 µm).<br>• ~15% of fibres are type I/ slow fibres, (likely within normal limits at this gestation).<br>• There is no size disparity between type I and II fibres, and there is a normal checkerboard distribution (no fibre type grouping).<br>• Approximately 10% of fibres have central nuclei.<br>• No cytoplasmic inclusions, clearing or rods.<br>• Oxidative enzyme stains (NADH, SDH and COX) have a normal uniform cytoplasmic staining pattern with focal peripheral accentuation and no cores or aggregates.

	• Subtle increase in the amount of fibrous tissue in quadriceps.<br>• Fibre type staining uninterpretable.	• Normal staining with acid phosphatase, with minimal punctate staining in the cytoplasm of the myocytes.<br>• A single vacuolated myocyte is seen, and rare enlarged nuclei, but no specific diagnostic cytological abnormalities.<br>• In the formalin fixed muscle including the diaphragm and intercostal muscles, there is no significant fibre size variation, and while the myofibres in the diaphragm are well defined, there is no fibrosis or fatty replacement. No evidence of fibre atrophy or loss is seen.
Musculoskeletal	• The limbs were thin and gracile, with mild gracility of the long bones and possible parietal Wormian bones.<br>• Cranial bones were normally formed.<br>• Congenital dislocation of the hips.<br>• Fixed flexion deformity<br>o Elbows mildly flexed.<br>o Wrists hyperextended and fixed.<br>o Fingers flexed, mainly at the metacarpophalangeal joints and also the proximal interphalangeal joints.<br>o Lower limbs showing fixed flexion deformities at the hips, knees and ankles.<br>o Bilateral clubfeet, relatively symmetrical and fixed.	• There are deformities of the upper and lower limbs.<br>• There is full range of motion of the shoulders, and the elbows are stiff but have a full range of motion. There is no flexion at the wrists, which have a range of motion of approximately 30 degrees of extension.<br>• The bilateral metacarpophalangeal joints are fixed at 90 degrees of flexion, and the proximal interphalangeal joints are flexed at 90 degrees with approximately 30 degrees of motion. The distal interphalangeal joints are fixed in extension. The thumbs are fixed to the palm in adduction. There are bilateral transverse palmar creases, and the distal phalangeal creases are absent.<br>• The hips are held in adduction, with limitation of abduction to approximately 70 degrees. There is normal flexion, extension and rotation of the hips.<br>• The knees have full range of motion.<br>• Both ankles are held in flexion at approximately 90 degrees and have approximately 30 degrees of range of motion.<br>• There are varus deformities of both feet and rocker bottom malformations.<br>• Eleven thoracic vertebral bodies and ribs (likely an incidental finding).

Table S4 Coding regions and canonical splice sites of the genes in the PCH panel

Gene	Coverage*
VRK1	100%
EXOSC3	100%
TSEN54	85.8%
TSEN2	99%
TSEN34	89.7%
SEPSECS	96%
RARS2	100%

* Sequenced with a mean depth of coverage of 148x with 93.4% of the target bases covered at least 20x.

Table S6 UFMylation proteins in 'Proteomics Dataset 1'

Gene Name	Uniprot ID	UFMylation process	Log2FC (AII-3 vs C4)	Q-score (*<0.05)
UFM1	P61960	Molecule	-0.08	0.0282*
UBA5	Q9GZZ9	Machinery	0.07	0.0208*
UFC1	Q9Y3C8	Machinery	0.01	0.731
DDRGK1	Q96HY6	Machinery	-0.2	0.000179*
UFL1	O94874	Machinery	-0.28	0.0000914*
UFSP2	Q9NUQ7	Machinery	0.08	0.171
**CDK5RAP3**	**Q96JB5**	**Scaffolding**	**-1.9**	**0.0000000186***
ODR4	Q5SWX8	Scaffolding	0.14	0.00363*
RPL26	J3QQQ9:P61254	Substrates	0.12	0.000293*
RPS20	P60866	Substrates	0.09	0.00387*
RPL10:RPL10L	P27635:Q96L21	Substrates	0.08	0.00338*
EIF6	P56537:A0A0U1RQV5	Substrates	0.34	0.00000381*
RPN1	P04843	Substrates	0.05	0.321
TP53	N/D	Substrates	N/D	N/D
MRE11	P49959	Substrates	-0.14	0.00381*
HIST1H4	N/D	Substrates	N/D	N/D
TRIP4	Q15650	Substrates	0.19	0.0000173*
CYB5R3	P00387:A0A087WZB1	Substrates	-0.01	0.801
HRD1	N/D	Substrates	N/D	N/D
SLC7A11	Q9UPY5	Substrates	0.36	0.00000449*
CD274	N/D	Substrates	N/D	N/D
**KIF11**	**P52732**	**Substrates**	**1.4**	**0.000341***
P4HB	H7BZ94	Substrates	-0.38	0.0000948*
P4HB	P07237	Substrates	-0.19	0.0000071*
ATG9A	Q7Z3C6:H7C152	Substrates	0.04	0.301

Table S12 UFMylation proteins in 'Proteomics Dataset 2'

Gene Name	Uniprot ID	UFMylation process	Log2FC (AII-3(T)	Q-score (*<0.05)

			vs AII-3(S))	
UFM1	P61960	Molecule	-0.15	0.0111*
UBA5	Q9GZZ9	Machinery	-0.036	0.253
UFC1	Q9Y3C8	Machinery	-0.089	0.0403*
DDRGK1	Q96HY6	Machinery	-0.1	0.0678
UFL1	O94874	Machinery	0.036	0.39
UFSP2	Q9NUQ7	Machinery	-0.06	0.039*
**CDK5RAP3**	**Q96JB5**	**Scaffolding**	**1.81**	**0.00000000214***
ODR4	Q5SWX8	Scaffolding	-0.032	0.384
RPL26	J3QQQ9:P61254	Substrates	-0.052	0.52
RPS20	P60866	Substrates	0.11	0.0859
RPL10:RPL10L	P27635:Q96L21	Substrates	-0.025	0.819
EIF6	P56537:A0A0U1RQV5	Substrates	-0.11	0.0128*
RPN1	P04843	Substrates	-0.059	0.252
TP53	N/D	Substrates	N/D	N/D
MRE11	P49959	Substrates	0.42	0.00736*
HIST1H4	N/D	Substrates	N/D	N/D
TRIP4	Q15650	Substrates	-0.018	0.662
CYB5R3	P00387:A0A087WZB1	Substrates	0.038	0.671
HRD1	N/D	Substrates	N/D	N/D
SLC7A11	Q9UPY5	Substrates	0.054	0.888
CD274	Q9NZQ7	Substrates	0.28	0.0142*
KIF11	N/D	Substrates	N/D	N/D
P4HB:PDIA2	P07237:Q13087	Substrates	0.032	0.673
ATG9A	Q7Z3C6:H7C152	Substrates	-0.15	0.13

Table S13 UFMylation proteins in 'Phosphoroteomics Dataset'

**Gene Name**	**Phospho ID**	**Phospho type**	**UFMylation process**	**Log2FC (AII-3(T) vs AII-3(S))**	**Q-score (*<0.05)**
UFM1	N/D	N/D	Molecule	N/D	N/D
UBA5	N/D	N/D	Machinery	N/D	N/D
UFC1	N/D	N/D	Machinery	N/D	N/D
DDRGK1	N/D	N/D	Machinery	N/D	N/D
UFL1	O94874|431	S	Machinery	-0.0062	0.956
UFL1	O94874|458	S	Machinery	0.22	0.0168*
**UFL1**	**O94874|462**	**S**	**Machinery**	**0.6**	**0.00126***
UFL1	O94874|789	S	Machinery	0.093	0.0251*
UFL1	O94874|792	T	Machinery	0.27	0.0193*
UFSP2	N/D	N/D	Machinery	N/D	N/D
CDK5RAP3	N/D	N/D	Scaffolding	N/D	N/D
ODR4	N/D	N/D	Scaffolding	N/D	N/D
RPL26	J3QQQ9:P61254|23:23	S	Substrates	-0.16	0.0647

RPS20	N/D	N/D	Substrates	N/D	N/D
RPL10:RPL10L	N/D	N/D	Substrates	N/D	N/D
EIF6	P56537|243	S	Substrates	-0.3	0.00347*
RPN1	N/D	N/D	Substrates	N/D	N/D
TP53	P04637|315	S	Substrates	-0.0074	0.928
MRE11	P49959|649	S	Substrates	0.44	0.00232*
HIST1H4	N/D		Substrates	N/D	N/D
TRIP4	Q15650|223	S	Substrates	-0.065	0.489
CYB5R3	P00387|146	S	Substrates	-0.03	0.823
CYB5R3	P00387|38	S	Substrates	0.027	0.8
HRD1	N/D	N/D	Substrates	N/D	N/D
**SLC7A11**	**Q9UPY5|26**	**S**	**Substrates**	**-0.59**	**0.00467***
CD274	Q9NZQ7|283	S	Substrates	0.27	0.0155*
KIF11	P52732|926	T	Substrates	-0.17	0.303
P4HB	P07237|32	S	Substrates	0.0027	0.991
ATG9A	Q7Z3C6:H7C152|656:72	S	Substrates	-0.058	0.182
**ATG9A**	**Q7Z3C6:H7C152|735:151**	**S**	**Substrates**	**-0.55**	**0.000208***
ATG9A	Q7Z3C6:H7C152|828:244	S	Substrates	-0.22	0.00398*

References

1 Al-Saady ML, Kaiser CS, Wakasuqui F, Korenke GC, Waisfisz Q, Polstra A, Pouwels PJW, Bugiani M, van der Knaap MS, Lunsing RJet al (2021) Homozygous UBA5 Variant Leads to Hypomyelination with Thalamic Involvement and Axonal Neuropathy. Neuropediatrics 52: 489-494. https://doi.org/10.1055/s-0041-1724130

2 Anazi S, Maddirevula S, Faqeih E, Alsedairy H, Alzahrani F, Shamseldin HE, Patel N, Hashem M, Ibrahim N, Abdulwahab Fet al (2017) Clinical genomics expands the morbid genome of intellectual disability and offers a high diagnostic yield. Mol Psychiatry 22: 615-624. https://doi.org/10.1038/mp.2016.113

3 Arnadottir GA, Jensson BO, Marelsson SE, Sulem G, Oddsson A, Kristjansson RP, Benonisdottir S, Gudjonsson SA, Masson G, Thorisson GAet al (2017) Compound heterozygous mutations in UBA5 causing early-onset epileptic encephalopathy in two sisters. BMC Med Genet 18: 103. https://doi.org/10.1186/s12881-017-0466-8

4 Benjamini Y, Hochberg Y (1995) Controlling the False Discovery Rate: A Practical and Powerful Approach to Multiple Testing. Journal of the Royal Statistical Society 57: 289-300.

5 Bergant G, Maver A, Peterlin B (2021) Whole-Genome Sequencing in Diagnostics of Selected Slovenian Undiagnosed Patients with Rare Disorders. Life (Basel) 11. https://doi.org/10.3390/life11030205

6 Briere LC, Walker MA, High FA, Cooper C, Rogers CA, Callahan CJ, Ishimura R, Ichimura Y, Caruso PA, Sharma Net al (2021) A description of novel variants and review of phenotypic spectrum in UBA5-related early epileptic encephalopathy. Cold Spring Harb Mol Case Stud 7. https://doi.org/10.1101/mcs.a005827

7 Cabrera-Serrano M, Coote DJ, Azmanov D, Goullee H, Andersen E, McLean C, Davis M, Ishimura R, Stark Z, Vallat JMet al (2020) A homozygous UBA5 pathogenic variant causes a fatal congenital neuropathy. J Med Genet 57: 835-842. https://doi.org/10.1136/jmedgenet-2019-106496

8 Cheema H, Bertoli-Avella AM, Skrahina V, Anjum MN, Waheed N, Saeed A, Beetz C, Perez-Lopez J, Rocha ME, Alawbathani Set al (2020) Genomic testing in 1019 individuals from 349 Pakistani families results in high diagnostic yield and clinical utility. NPJ Genom Med 5: 44. https://doi.org/10.1038/s41525-020-00150-z

9 Chen H, LaFlamme CW, Wang YD, Blan AW, Koehler N, Mendonca Moraes R, Olszewski AR, Almanza Fuerte EP, Bonkowski ES, Bajpai Ret al (2025) Patient-derived models of UBA5-associated encephalopathy identify defects in neurodevelopment and highlight potential therapeutic avenues. Sci Transl Med 17: eadn8417. https://doi.org/10.1126/scitranslmed.adn8417

10 Chitre M, Nahorski MS, Stouffer K, Dunning-Davies B, Houston H, Wakeling EL, Brady AF, Zuberi SM, Suri M, Parker APJet al (2018) PEHO syndrome: the endpoint of different genetic epilepsies. J Med Genet 55: 803-813. https://doi.org/10.1136/jmedgenet-2018-105288

11 Colin E, Daniel J, Ziegler A, Wakim J, Scrivo A, Haack TB, Khiati S, Denomme AS, Amati-Bonneau P, Charif Met al (2016) Biallelic Variants in UBA5 Reveal that Disruption of the UFM1 Cascade Can Result in Early-Onset Encephalopathy. Am J Hum Genet 99: 695-703. https://doi.org/10.1016/j.ajhg.2016.06.030

12 Cox J, Mann M (2008) MaxQuant enables high peptide identification rates, individualized p.p.b.-range mass accuracies and proteome-wide protein quantification. Nat Biotechnol 26: 1367-1372. https://doi.org/10.1038/nbt.1511

13 Daida A, Hamano SI, Ikemoto S, Matsuura R, Nakashima M, Matsumoto N, Kato M (2018) Biallelic loss-of-function UBA5 mutations in a patient with intractable West syndrome and profound failure to thrive. Epileptic Disord 20: 313-318. https://doi.org/10.1684/epd.2018.0981

14 Di Rocco M, Rusmini M, Caroli F, Madeo A, Bertamino M, Marre-Brunenghi G, Ceccherini I (2018) Novel spondyloepimetaphyseal dysplasia due to UFSP2 gene mutation. Clin Genet 93: 671-674. https://doi.org/10.1111/cge.13134
15 Duan R, Shi Y, Yu L, Zhang G, Li J, Lin Y, Guo J, Wang J, Shen L, Jiang Het al (2016) UBA5 Mutations Cause a New Form of Autosomal Recessive Cerebellar Ataxia. PLoS One 11: e0149039. https://doi.org/10.1371/journal.pone.0149039
16 Egunsola AT, Bae Y, Jiang MM, Liu DS, Chen-Evenson Y, Bertin T, Chen S, Lu JT, Nevarez L, Magal Net al (2017) Loss of DDRGK1 modulates SOX9 ubiquitination in spondyloepimetaphyseal dysplasia. J Clin Invest 127: 1475-1484. https://doi.org/10.1172/JCI90193
17 Franceschi R, Iascone M, Maitz S, Marchetti D, Mariani M, Selicorni A, Soffiati M, Maines E (2022) A missense mutation in DDRGK1 gene associated to Shohat-type spondyloepimetaphyseal dysplasia: Two case reports and a review of literature. Am J Med Genet A 188: 2434-2437. https://doi.org/10.1002/ajmg.a.62857
18 Gagnon-Bartsch JA, Speed TP (2012) Using control genes to correct for unwanted variation in microarray data. Biostatistics 13: 539-552. https://doi.org/10.1093/biostatistics/kxr034
19 Glinos DA, Garborcauskas G, Hoffman P, Ehsan N, Jiang L, Gokden A, Dai X, Aguet F, Brown KL, Garimella Ket al (2022) Transcriptome variation in human tissues revealed by long-read sequencing. Nature 608: 353-359. https://doi.org/10.1038/s41586-022-05035-y
20 Hamilton EMC, Bertini E, Kalaydjieva L, Morar B, Dojcakova D, Liu J, Vanderver A, Curiel J, Persoon CM, Diodato Det al (2017) UFM1 founder mutation in the Roma population causes recessive variant of H-ABC. Neurology 89: 1821-1828. https://doi.org/10.1212/WNL.0000000000004578
21 Han Y, Ge Y, Liu H, Liu L, Xie L, Chen X, Chen Q (2024) A novel compound heterozygous mutation of UFC1 in a patient with neurodevelopmental disorder. Genes Genomics 46: 1037-1043. https://doi.org/10.1007/s13258-024-01543-5
22 Huber W, von Heydebreck A, Sultmann H, Poustka A, Vingron M (2002) Variance stabilization applied to microarray data calibration and to the quantification of differential expression. Bioinformatics 18 Suppl 1: S96-104. https://doi.org/10.1093/bioinformatics/18.suppl_1.s96
23 Ivanov I, Pacheva I, Yordanova R, Sotkova I, Galabova F, Gaberova K, Panova M, Gheneva I, Tsvetanova T, Noneva Ket al (2023) Hypomyelination with Atrophy of Basal Ganglia and Cerebellum (HABC) Due to UFM1 Mutation in Roma Patients - Severe Early Encephalopathy with Stridor and Severe Hearing and Visual Impairment. A Single Center Experience. CNS Neurol Disord Drug Targets 22: 207-214. https://doi.org/10.2174/1871527321666220221100704
24 Low KJ, Baptista J, Babiker M, Caswell R, King C, Ellard S, Scurr I (2019) Hemizygous UBA5 missense mutation unmasks recessive disorder in a patient with infantile-onset encephalopathy, acquired microcephaly, small cerebellum, movement disorder and severe neurodevelopmental delay. Eur J Med Genet 62: 97-102. https://doi.org/10.1016/j.ejmg.2018.06.009
25 Mattern L, Begemann M, Delbruck H, Holschbach P, Schroder S, Schacht SM, Kurth I, Elbracht M (2023) Variant of the catalytic cysteine of UFSP2 leads to spondyloepimetaphyseal dysplasia type Di Rocco. Bone Rep 18: 101683. https://doi.org/10.1016/j.bonr.2023.101683
26 Mignon-Ravix C, Milh M, Kaiser CS, Daniel J, Riccardi F, Cacciagli P, Nagara M, Busa T, Liebau E, Villard L (2018) Abnormal function of the UBA5 protein in a case of early developmental and epileptic encephalopathy with suppression-burst. Hum Mutat 39: 934-938. https://doi.org/10.1002/humu.23534
27 Miller CR, Lee K, Pfau RB, Reshmi SC, Corsmeier DJ, Hashimoto S, Dave-Wala A, Jayaraman V, Koboldt D, Matthews Tet al (2020) Disease-associated mosaic variation in clinical exome sequencing: a two-year pediatric tertiary care experience. Cold Spring Harb Mol Case Stud 6. https://doi.org/10.1101/mcs.a005231

28 Molania R, Gagnon-Bartsch JA, Dobrovic A, Speed TP (2019) A new normalization for Nanostring nCounter gene expression data. Nucleic Acids Res 47: 6073-6083. https://doi.org/10.1093/nar/gkz433

29 Muona M, Ishimura R, Laari A, Ichimura Y, Linnankivi T, Keski-Filppula R, Herva R, Rantala H, Paetau A, Poyhonen Met al (2016) Biallelic Variants in UBA5 Link Dysfunctional UFM1 Ubiquitin-like Modifier Pathway to Severe Infantile-Onset Encephalopathy. Am J Hum Genet 99: 683-694. https://doi.org/10.1016/j.ajhg.2016.06.020

30 Nahorski MS, Maddirevula S, Ishimura R, Alsahli S, Brady AF, Begemann A, Mizushima T, Guzman-Vega FJ, Obata M, Ichimura Yet al (2018) Biallelic UFM1 and UFC1 mutations expand the essential role of ufmylation in brain development. Brain 141: 1934-1945. https://doi.org/10.1093/brain/awy135

31 Ni M, Afroze B, Xing C, Pan C, Shao Y, Cai L, Cantarel BL, Pei J, Grishin NV, Hewson Set al (2021) A pathogenic UFSP2 variant in an autosomal recessive form of pediatric neurodevelopmental anomalies and epilepsy. Genet Med 23: 900-908. https://doi.org/10.1038/s41436-020-01071-z

32 Otaify GA, Al Baluki W, Al-Rashdi S, Al-Maawali A (2022) Shohat type-spondyloepimetaphyseal dysplasia: Further phenotypic delineation. Eur J Med Genet 65: 104640. https://doi.org/10.1016/j.ejmg.2022.104640

33 Papuc SM, Abela L, Steindl K, Begemann A, Simmons TL, Schmitt B, Zweier M, Oneda B, Socher E, Crowther LMet al (2019) The role of recessive inheritance in early-onset epileptic encephalopathies: a combined whole-exome sequencing and copy number study. Eur J Hum Genet 27: 408-421. https://doi.org/10.1038/s41431-018-0299-8

34 Raha S, Kotecha U, Mistri M, Shah P, Sharda S (2023) Familial Infantile spasm syndrome due to biallelic variants in the gene encoding UFM1-specific peptidase 2 (UFSP2). Medical Reports 2 100002. https://doi.org/https://doi.org/10.1016/j.hmedic.2023.100002

35 Storey JD (2002) A direct approach to false discovery rates. Journal of the Royal Statistical Society Series B (Statistical Methodology): 479 - 498.

36 Szucs Z, Fitala R, Nyuzo AR, Fodor K, Czemmel E, Vrancsik N, Bessenyei M, Szabo T, Szakszon K, Balogh I (2021) Four New Cases of Hypomyelinating Leukodystrophy Associated with the UFM1 c.-155_-153delTCA Founder Mutation in Pediatric Patients of Roma Descent in Hungary. Genes (Basel) 12. https://doi.org/10.3390/genes12091331

37 Tsang E, Han VX, Flutter C, Alshammery S, Keating BA, Williams T, Gloss BS, Graham ME, Aryamanesh N, Pang Iet al (2024) Ketogenic diet modifies ribosomal protein dysregulation in KMT2D Kabuki syndrome. EBioMedicine 104: 105156. https://doi.org/10.1016/j.ebiom.2024.105156

38 Watson CM, Crinnion LA, Gleghorn L, Newman WG, Ramesar R, Beighton P, Wallis GA (2015) Identification of a mutation in the ubiquitin-fold modifier 1-specific peptidase 2 gene, UFSP2, in an extended South African family with Beukes hip dysplasia. S Afr Med J 105: 558-563. https://doi.org/10.7196/SAMJnew.7917

39 Zaman Z, Straka N, Pinto AL, Srouji R, Tam A, Periasamy U, Stone S, Kleinman M, Northam WT, Ebrahimi-Fakhari D (2023) Deep brain stimulation for medically refractory status dystonicus in UBA5-related disorder. Mov Disord 38: 1757-1759. https://doi.org/10.1002/mds.29428

40 Zech M, Dzinovic I, Skorvanek M, Harrer P, Necpal J, Kopajtich R, Kittke V, Tilch E, Zhao C, Tsoma Eet al (2025) Combined genomics and proteomics unveils elusive variants and vast aetiologic heterogeneity in dystonia. Brain 148: 2827-2846. https://doi.org/10.1093/brain/awaf059

41 Zhang G, Tang S, Wang H, Pan H, Zhang W, Huang Y, Kong J, Wang Y, Gu J, Wang Y (2020) UFSP2-related spondyloepimetaphyseal dysplasia: A confirmatory report. Eur J Med Genet 63: 104021. https://doi.org/10.1016/j.ejmg.2020.104021

42 Zhou Y, Zhou B, Pache L, Chang M, Khodabakhshi AH, Tanaseichuk O, Benner C, Chanda SK (2019) Metascape provides a biologist-oriented resource for the analysis of systems-level datasets. Nat Commun 10: 1523. https://doi.org/10.1038/s41467-019-09234-6
